# Enhanced Sensitivity to Local Dynamics in Peptides by Use of Temperature‐Jump IR Spectroscopy and Isotope Labeling

**DOI:** 10.1002/chem.201904497

**Published:** 2020-02-04

**Authors:** David Scheerer, Heng Chi, Dan McElheny, Timothy A. Keiderling, Karin Hauser

**Affiliations:** ^1^ Department of Chemistry University of Konstanz 78457 Konstanz Germany; ^2^ Department of Chemistry University of Illinois at Chicago Chicago IL USA; ^3^ Jiangsu Food and Pharmaceutical Science College Huai'an P.R. China

**Keywords:** isotopic labeling, molecular dynamics, peptides, time-resolved spectroscopy, vibrational coupling

## Abstract

Site‐specific isotopic labeling of molecules is a widely used approach in IR spectroscopy to resolve local contributions to vibrational modes. The induced frequency shift of the corresponding IR band depends on the substituted masses, as well as on hydrogen bonding and vibrational coupling. The impact of these different factors was analyzed with a designed three‐stranded β‐sheet peptide and by use of selected ^13^C isotope substitutions at multiple positions in the peptide backbone. Single‐strand labels give rise to isotopically shifted bands at different frequencies, depending on the specific sites; this demonstrates sensitivity to the local environment. Cross‐strand double‐ and triple‐labeled peptides exhibited two resolved bands that could be uniquely assigned to specific residues, the equilibrium IR spectra of which indicated only weak local‐mode coupling. Temperature‐jump IR laser spectroscopy was applied to monitor structural dynamics and revealed an impressive enhancement of the isotope sensitivity to both local positions and coupling between them, relative to that of equilibrium FTIR spectroscopy. Site‐specific relaxation rates were altered upon the introduction of additional cross‐strand isotopes. Likewise, the rates for the global β‐sheet dynamics were affected in a manner dependent on the distinct relaxation behavior of the labeled oscillator. This study reveals that isotope labels provide not only local structural probes, but rather sense the dynamic complexity of the molecular environment.

## Graphical abstract

**Enhanced sensitivity**: Site‐selected dynamics of a three‐stranded β‐sheet peptide are probed by laser‐excited temperature‐jump infrared spectroscopy and isotopic labeling. Relaxation time constants show an enhanced sensitivity to vibrational coupling.

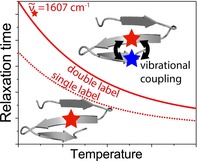

## Introduction

Protein activity is intimately linked to local structure and dynamics, for example, by positioning functional groups or substrates in enzymes, thereby allowing the structure to execute biochemical processes. A comprehensive view of protein‐folding mechanisms requires an understanding of dynamics and structure.^1^ Determining how a protein achieves such a functional and stable, low‐energy state is at the core of both the overall protein‐folding problem and growing interest in misfolded proteins, which become trapped in even lower energy minima. Many degenerative diseases have their pathology rooted in protein misfolding and aggregation, which often involves conformational transitions to β‐sheet structures.^2^ Thus, delineating the stabilizing interactions and factors that affect conformational dynamics of β‐sheets is of particular interest in biophysical research on protein folding, misfolding, and aggregation. Determination of the protein backbone structure, or fold, and its dynamics requires methods that sense the coupling of specific peptide units, which form the fundamental polymer chain and that are capable of a relatively fast response upon structural changes.

Vibrational spectroscopy provides a useful means for attaining molecular‐scale insights into structural and dynamic properties of proteins. IR spectra are particularly beneficial in analyzing β‐sheet structures.^3^ Global secondary structure changes can be monitored with equilibrium and dynamic techniques by probing the amide I band, which involves the C=O stretching modes of the polypeptide backbone. By contrast, electronic circular dichroism (ECD) in the UV region, *λ*>200 nm, is weak for sheet structures,^4^ and fluorescence mainly detects changes in tertiary structures. Nonetheless, optical spectra are low resolution and lack site‐specific sensitivity to local structural variations. However, by the use of isotopic substitution, contributions of individual modes can be resolved and identified in both equilibrium and dynamic vibrational spectroscopic studies.^5^ In particular, the substitution of selected amide C=O groups with ^13^C leads to IR amide I frequencies downshifted by about 40 cm^−1^ from where they would appear in the spectrum if isolated as a ^12^C=O mode. The properties of such isolated ^13^C=O modes can be attributed to local conformations and their dynamic changes.^5^, ^6^ It should be noted that frequency shifts for single‐labeled peptides alone are not sufficient to determine local structure, but their changes upon unfolding can be diagnostic, particularly if the stable structure is determined independently with other techniques. If two or more such labeled residues are incorporated into the sequence, their specific coupling can be exploited to better determine local structure; this is often aided by theoretical modeling.^7^

Beyond structure, isotopic substitution provides a means of studying site‐specific, fast dynamics of β‐sheets that can be accessed using laser‐induced temperature‐jump (T‐jump) spectroscopy with tunable single‐wavelength IR detection.^8^ Herein, we used T‐jump IR techniques to gain new insights into the spectroscopic and folding properties of isotopically labeled β‐sheet model peptides. Many studies have utilized sequence designs with cross‐strand aromatic interactions^9^ and/or turn‐promoting sequences^10^ to initialize the formation of β‐hairpin structures. Previously, we reported spectra and dynamics for a series of three‐stranded double hairpin designs8g, 8i based on the very stable ^D^Pro−Gly turn sequence.8i, 10b, 11 However, its strong structural constraints can overemphasize the role of turns in the folding mechanism.^12^ Substituting the turns with Aib−Gly (Aib=α‐aminoisobutyric acid) sequences, following the designs of Hammer and co‐workers,7b, 13 can relieve some of these conformational constraints and eliminate spectral overlap of ^13^C=O‐labeled peptide modes with those of the Xxx−Pro peptide link, while still promoting turn formation in aqueous solution. The sequences we study herein form three‐stranded sheets, the central strands of which are hydrogen bonded to both the first and third strands; this mimics interactions in a more extended sheet structure. We additionally incorporated a Trp−Tyr cross‐strand interaction between the first two strands to preferentially stabilize that hairpin, following previous studies.8i, 9a We analyzed the impact of various labeling schemes on IR spectra and, in particular, on T‐jump‐induced relaxation rates. Results were compared with complementary quantum mechanical spectral calculations, NMR structures, and molecular dynamics (MD) simulations. Unexpectedly, we observed only minor effects of cross‐strand coupling for labeled residues in the equilibrium IR spectra, whereas the site‐selected T‐jump‐induced kinetics obtained with isotope‐labeled probes had significantly enhanced sensitivity to coupling.

## Experimental Section

**Scheme 1:**
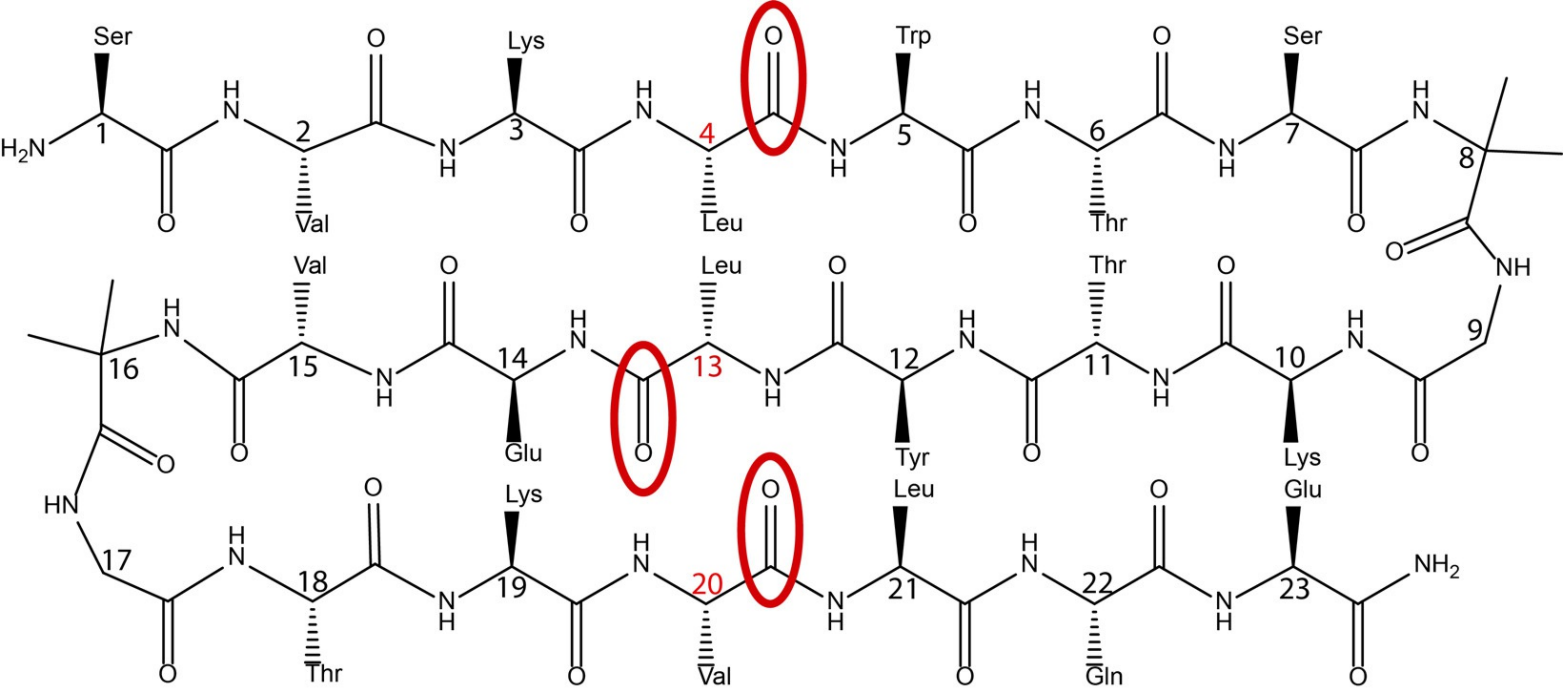
Generic layout of the three‐stranded sheet model with the isotopically labeled positions—Leu4, Leu13, and Val20—indicated by red ellipses. Note that the peptide variants studied herein contain either single, double, or triple labels.

The sequence of the peptides used in this study (SVKLWTS‐BG‐KTYLEV‐BG‐TKVLQE‐NH_2_; B=Aib^13^) was modeled after a design of Gellman,^14^ as discussed previously,8i and is illustrated in Scheme 1. Substitution of Aib−Gly for the ^D^Pro−Gly turn sequence used by Gellman was suggested by our earlier demonstration that the Aib−Gly sequence supported hairpin formation.11c, 13, 15 Isotopic labels, ^13^C on the amide C=O, were introduced at specific positions roughly centered on the three β‐strands of the peptide (at Leu4, Leu13, and Val20). These substitutions were chosen to enable cross‐strand multiple labeling that could potentially increase the intensities of the shifted bands. It is clear from the *R* ^−3^ dependence of dipole coupling that placing labels close within the sequence leads to stronger coupling, but for opposing strands this can be more complex. We previously found that forming small cross‐strand hydrogen‐bonded rings in hairpin structures led to significant coupling and herein incorporated that into our design.8d, 13, 16 Label placement provided a test for coupling across strands 1–2, for labels on residues Leu4–Leu13; across strand 2–3, for labels on Leu13–Val20; and, as a comparison, labeled on all three strands. The peptides were named according to the label positions. Consequently, the single‐label variants were **1W‐4**, **1W‐13**, and **1W‐20**; the double‐labeled variants were **1W‐4**–**13** and **1W‐13**–**20**, and the corresponding triple‐labeled variant was **1W‐4**–**13**–**20**. Additional residues, including Gly9, Val15, Gly17, and Leu21, were labeled and investigated using FTIR spectroscopy for further testing of the computed model force field (FF).

### Peptide sample preparation and FTIR spectroscopy

Peptides were obtained from SciLight Biotechnology LLC, Beijing, P.R. China, after being synthesized by using standard fluorenylmethoxycarbonyl (FMOC) methods. Sample purity in each case was judged as >95 % based on MS and HPLC analyses. Sample preparation and temperature‐dependent equilibrium IR spectra for all isotopically labeled variants were obtained, as described previously.8i Further experimental details are also given in the Supporting Information. Temperature‐dependent IR spectra were measured over the temperature range of 5–95 °C in steps of Δ*T*=5 °C, and the results were analyzed by using singlevalue decomposition (SVD) methods. The peptides were dissolved in D_2_O at ∼10 mg mL^−1^ at acidic pH and lyophilized threefold to remove trifluoroacetic acid (TFA) and effect H/D exchange before being placed in a homemade demountable cell consisting of CaF_2_ windows with a Teflon spacer (100 μm optical path length). Samples for T‐jump experiments were prepared similarly.

### T‐jump relaxation dynamics

Relaxation kinetics were obtained by using the laser spectrometer we have described in detail separately.8h, 12 In brief, a pulsed Q‐switched Ho:YAG laser (IPG Photonics Corporation, USA) operated at *λ*=2090 nm was used to excite a solvent (D_2_O) vibration, thereby rapidly increasing the sample temperature. To improve uniform sample heating, the pump beam was split into two counter‐propagating beams, both focused on the sample, with one delayed, leading to an effective pulse duration of ∼15 ns. The rapid T‐jump perturbs the folding equilibrium on a timescale faster than that of the molecular dynamics of interest. A chopper was synchronized with the 10 Hz pump laser and blocked each second pulse to provide a reference signal with no excitation, which resulted in an excitation repetition rate of 5 Hz. The pump energy was set to a maximum of 14 mJ to yield a T‐jump magnitude of ∼8 °C; however, for experiments at the lowest final temperatures of ∼5 °C, a smaller jump was required and obtained by reducing the pump power with neutral density attenuators. Relaxation dynamics of the peptide were probed at selected wavenumbers with a quantum cascade laser (QCL), installed in a MIRcat‐QT laser system (Daylight Solutions Inc., USA). The continuous‐wave (cw) QCL used (M2062‐PCX) had a tuning range from 1730 to 1480 cm^−1^, which was ideally suited for the ^13^C=O isotope studies. The probe laser beam was focused on the sample within a spot (*Ø* ∼300 μm) that was significantly smaller than that of the excitation beams (*Ø*∼2 mm), to assure measurement of a homogenously heated volume.

To correct for the influence of solvent kinetics, the sequentially measured solvent‐only signal was scaled and subtracted from the peptide sample signal to result in a flat baseline after completion of peptide relaxation. The final temperature after the T‐jump was determined from the change in absorbance of the solvent, which was measured under the same pumping conditions, and by calibration with temperature‐dependent FTIR spectra of D_2_O as a reference.8g Relaxation kinetics for ∼1000 transients were averaged and evaluated for the time interval from 300 ns up to 1.2 ms by using a monoexponential decay function. Time constants, *τ*, were determined for different final temperatures, varying from 5 to 50 °C, and the resultant rate constants, *k*, were fit to an Arrhenius relationship.

### Structures determined by NMR spectroscopy

An ensemble of best‐fit low‐energy structures was determined for a closely related peptide (SVKIWTS‐BG‐KTYTEV‐BG‐TKTLQE‐NH_2_) by analysis of 2D NOESY, TOCSY, and COSY NMR spectra. The use of structural data for this alternate sequence was supported by higher solubility, very similar chemical shifts in both sequences for the conserved residues, and the fact that the circular dichroism (CD) and IR spectra, as well as T‐jump kinetics of this alternate sequence and that used for the T‐jump studies, were almost identical. Structures were obtained for the peptide at 283 K in 90:10 H_2_O/D_2_O, at ∼6 mg mL^−1^ concentration (∼2–3 mm), by using an 800 MHz instrument with the same methods as those detailed previously.8i, 12 Spectra were processed within NMRPipe,^17^ viewed/assigned in NMRView,^18^ and NOESY signals were manually selected and assigned with CYANA 2.0.^19^ Data regarding NMR results and structure determination are available in Table S1 in the Supporting Information. The 10 lowest energy unique structures were refined by restrained MD within AMBER8,^20^ by using the ff99sb FF,^21^ and were used to guide our subsequent spectral simulations and analyses.

### Molecular dynamics

Simulations were carried out on the lowest energy NMR spectroscopy structure, which was obtained as described above. Briefly, the peptide was solvated in a box of TIP3P water, energy minimized, and annealed in a multistep process, as detailed previously.^12^ Unrestrained 200 ns NPT MD trajectories were carried out at 300 K by using the Amber FF FF14SB. CPPTRAJ^22^ was used to analyze the trajectories for information such as variation of torsional angles in turn residues and interatomic distances between strands for selected hydrogen bonds.

### Spectral computations

To provide some measure of spectral sensitivity to structure variations, spectral simulations for a set of 23‐residue all‐Ala peptides, each constrained to selected conformations, as determined by NMR spectroscopy (see above), were carried out at the DFT level (BPW91/6‐31G**/PCM) using Gaussian 16.^23^ The methods used closely followed our previous study,^12^ and are detailed in the Supporting Information. To account for the differences in central and outer‐strand hydration effects, DFT force fields (FF) were empirically adjusted to better reflect experimental frequency shifts of single‐labeled variants.

## Results and Discussion

### Structural aspects

The introduction of ^13^C=O backbone isotope labels into β‐sheet structures maximizes the potential for structural analysis by IR spectroscopy, if they are strongly coupled. Ideally, one might seek to design a peptide that forms three antiparallel strands fully hydrogen bonded to each other and interconnected by tight turns, which result in only a moderate twist of the overall structure. DFT simulations of a fully minimized, unrestrained three‐stranded structure show significant intensity and frequency changes for double‐labeled peptides that are indicative of strong coupling between strands. For the strongest coupling cases, the amide I′ (I′ indicates H–D exchanged) modes, corresponding to the single labels, were at similar frequencies. The predicted IR spectra for labeled variants of such a near‐ideal structure are illustrated in Figure S2 in the Supporting Information.

This design goal was approached in our previous study of the **pG2** peptide, but the ^D^Pro−Gly turn sequence, in that case, led to spectral interferences that inhibited interpretation of the impact of isotopic labeling on strand dynamics.^12^ To avoid this interference, we converted related sequences8i to incorporate Aib−Gly (BG) turns, which also support hairpin formation in water, as previously demonstrated with two‐strand models.7b, 11c, 13 Our initially prepared Aib−Gly variant incorporated an aromatic cross‐strand contact in the first, strand 1–2, hairpin (SVKIWTS‐BG‐KTYTEV‐BG‐TKTLQE‐NH_2_) and resulted in a stable three‐strand sheet structure, as confirmed by NMR structure determination, as well as IR and CD spectra. This sequence was subsequently mutated, I4→L4, T13→L13, and T20→V20, to yield SVKLWTS‐BG‐KTYLEV‐BG‐TKVLQE‐NH_2_, which allowed easier isotopic labeling with only minor effects on the structure. The similarity of their folds was shown by IR and CD results, as well as T‐jump kinetics, which were virtually the same for both sequences, although the revised sequence was somewhat more stable (based on temperature‐dependent IR).

**Figure 1:**
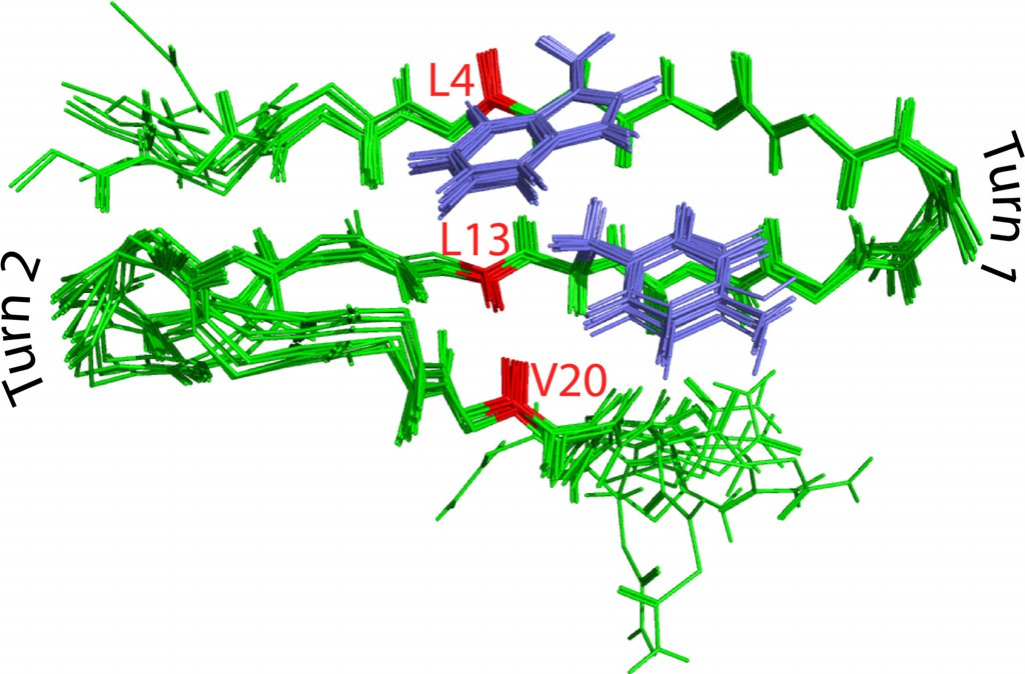
Overlay of the main chains of the 10 lowest energy solution NMR structures for SVKIWTS‐BG‐KTYTEV‐BG‐TKTLQE‐NH_2_. ^13^C=O oscillators are marked in red. Although all side chains are removed for clarity, the Trp and Tyr aromatic side chains, which have an offset stacking interaction, are shown highlighted in blue because they stabilize strands 1 and 2.

The NMR‐ determined structures show that this modified peptide is quite twisted, and overlapping the ensemble of the best‐fit structures suggests that the termini are disordered (Figure 1). The first turns (Aib8–Gly9) are quite uniform for the 10 best‐fit structures, but the second turns (Aib16–Gly17) vary more or less continuously between two extrema. Turn 2 does maintain *ϕ*,*ψ* torsions representative of type 1′ turns (see Table S3 in the Supporting Information), unlike the two‐state structures found for the ^D^Pro−Gly turns in our previous papers.8i, 12 The apparent variations in turn 2 are thus not local, but are a consequence of small deviations from the mean positions in their neighboring residues.

The 300 K MD analyses indicate somewhat different structural variations, but also support the relative stability of the turns, which have only short‐lived deviations away from type 1′. Similarly, the inner parts of the three‐stranded structure, in which the ^13^C=O labels are placed, remain folded in the MD simulations, whereas the termini are highly dynamic, as observed from differences in average hydrogen‐bond distances (Table S4 in the Supporting Information). The NMR data imply that the C terminus has somewhat more disorder than that of the N terminus and that the hairpin between strands 1 and 2 is more complete and uniform, on average. This appears to be a direct consequence of the cross‐strand stabilization induced by the aromatic Trp−Tyr interaction (of residues 5 and 12), which is very stable in both NMR and MD results. In contrast to the edge‐to‐face Trp−Tyr geometry observed for the tryptophan zipper (Trpzip) variants,9a, 9b, 16, 24 the aromatic interaction in this peptide is more of an offset stacking arrangement, similar to our previous ^D^Pro−Gly turn‐based studies.8i Clearly, in the actual structure, the N‐ and C‐terminal strands are not equivalent because both are distorted by disorder; but the hairpin formed with strands 1–2 is quite regular aside from the N‐terminal residues, as evident from the overlaid structures in Figure 1. Thus, one anticipates coupling between residues in these strands. The other hairpin between strands 2 and 3 is less well formed and the consequences for coupling are more difficult to predict. Finally, the computed MD trajectories also show large motions for the terminal residues that suggest use of a single structure for spectral simulation is likely to be incomplete at best, which has led us to compare spectral simulations for selected structures derived from the NMR spectroscopy best‐fit set.

### Shifting IR frequencies by isotopic labeling

**Figure 2:**
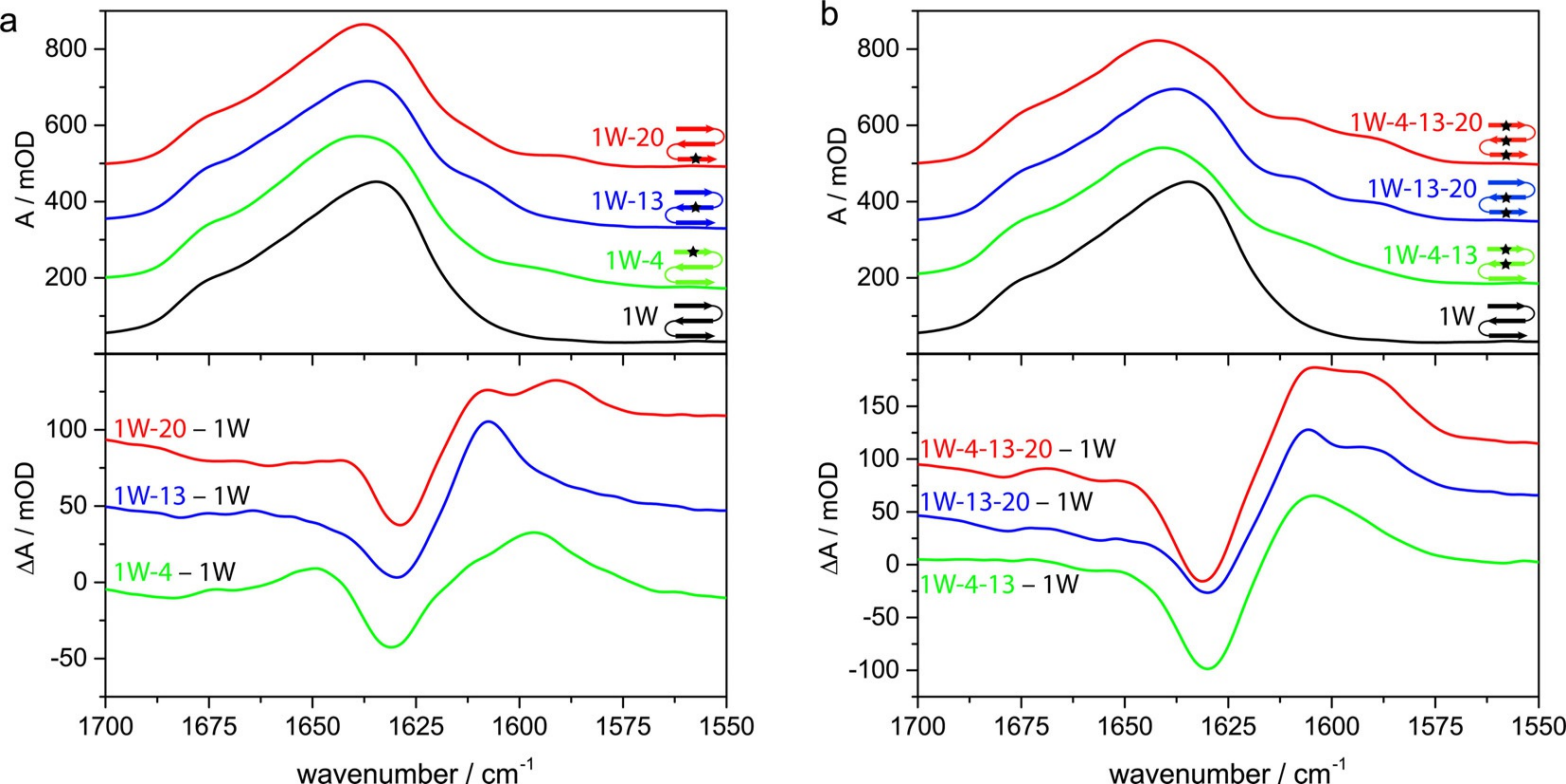
A comparison of the IR absorption spectra of the amide I′ band (top) and difference spectra in which the unlabeled variant is used as a reference subtracted from the others (bottom): a) Single‐labeled variants **1W‐4** (green), **1W‐13** (blue), and **1W‐20** (red) in comparison with the unlabeled variant **1W** (black). b) Multiple‐labeled variants **1W‐4**–**13** (green), **1W‐13**–**20** (blue), and **1W‐4**–**13**–**20** (red) in comparison to the unlabeled variant **1W** (black). The spectra were acquired in D_2_O at 10 °C and normalized to the band integral between 1700 and 1570 cm^−1^. Difference spectra were obtained by subtracting the normalized absorbance signal of the unlabeled variant to better show the presence, relative intensities, and positions of the labeled modes. Data were offset by 150 (top) and 50 mOD (bottom) for better visualization.

The low‐temperature IR spectra of all peptide variants (Figure 2) exhibited characteristics of antiparallel β‐sheets. For the unlabeled variant, **1W**, this is indicated by a major band at 1634 cm^−1^, with a weaker shoulder at ∼1674 cm^−1^.

Upon heating, the IR bands broaden and shift their maxima to 1648–1652 cm^−1^, which indicates the formation of a disordered state (Figure S5 in the Supporting Information). The very gradual transition indicated a low level of cooperativity with an apparent *T*_m_ of ∼73 °C, which was determined by fitting the derivative of the SVD second component versus temperature plot for the amide I′ band shape (Figure S6 in the Supporting Information).^25^

As expected, the introduction of isotopic labels gave rise to additional bands at lower wavenumbers (Figure 2 a and Figure S5 in the Supporting Information), the center frequencies of which are listed in Table 1. (Some additional isotopically labeled variants were prepared, for which results are given in Figure S7 in the Supporting Information.) In contrast to expectations for an ideal structure, in which local amide frequencies in each strand are the same, the frequencies of these bands show a strong dependency on the location of the oscillator within the β‐sheet. If the central strand (Leu13) is labeled, a shoulder at 1607 cm^−1^ is observed, but if the peptide is labeled on outer strands, lower wavenumber bands arise. Furthermore, the orientation of the labeled group has an impact. Val20, with its ^13^C=O pointing in toward the central strand, leads to a band at 1588 cm^−1^, whereas Leu4, with ^13^C=O pointing out toward the solvent, has a higher wavenumber band at 1594 cm^−1^. (By comparison, Gly9 and Gly17 also point out and, if labeled, have bands at 1591 cm^−1^ and 1592 cm^−1^, see Figure S7 in the Supporting Information; this suggests a measurable difference between internal hydrogen bonding to amides and solvation by water.) This lack of degeneracy for the various isotope positions reduces the impact of their mutual cross‐strand coupling. Labeled Val20 (and possibly also Leu4) gives rise to an additional minor side band at ∼1610 cm^−1^, which is most evident in the difference spectra (Figure 2). The side band related to Val20 is reproducible, which is consistent between peptides with different labeling patterns and is not attributable to impurities, as shown by the MS and HPLC results, as well as its appearing in the spectra of each of the labeled samples containing Val20. We do not have a conclusive assignment of this observed feature. However, we can suggest that it arises from conformational equilibria that encompass more structural variation in the C‐terminal residues than in the N‐terminal ones, which will affect Val20 more than Leu4.

**Table 1:** Maxima in cm^−1^ of ^13^C=O and β‐strand bands from the IR absorption spectra.

Peptide	Low‐frequency ^13^C=O	High‐frequency ^13^C=O	β‐Sheet
**1W**	n.a.^[a]^	n.a.^[a]^	1634
**1W‐4**	1594	n.a.^[a]^	1639
**1W‐13**	n.a.^[a]^	1607	1635
**1W‐20**	1588	n.a.^[a]^	1637
**1W‐4**–**13**	1587	1610	1641
**1W‐13**–**20**	1588	1608	1638
**1W‐4**–**13**–**20**	1588	1608	1642

[a] Not applicable.

### Mixing of IR modes

The double‐labeled variants **1W‐4**–**13** and **1W‐13**–**20** (see Figure 2 b) both show two distinct bands at ∼1608 and 1588 cm^−1^, which can be attributed to the labeled Leu13 in both and Leu4 or Val20 in each, respectively. This behavior is different from that observed in our previous studies of hairpins with cross‐strand labels. Those peptides had more strongly coupled transitions, for which the two isotope‐shifted modes generated overlapping bands with quite different intensities, the individual contributions of which could not be distinguished.7b, 8d, 13 The nondegeneracy seen here obscures coupling, and the roughly equivalent intensity in both bands implies that it is weak. However, the labeled oscillators are coupled, to some extent, as witnessed by shifts of the double‐label bands in **1W‐4**–**13** up and down in frequency from that found in **1W‐4** and **1W‐13**, respectively, can be observed from the values in Table 1. The shift of Leu13 (∼3 cm^−1^) is less than that for Leu4 (∼7 cm^−1^) in **1W‐4**–**13**, which is asymmetric, but substantial. By contrast, it is much less (∼1 cm^−1^) for Leu13 and Val20 in **1W‐13**–**20**, which suggests a difference in coupling for stands 1–2 and 2–3, the latter being much weaker. The spectrum of the triple‐labeled variant **1W‐4**–**13**–**20** appears to be a combination of the respective single‐ and double‐labeled sequences, resulting in a stronger intensity for the band at ∼1588 cm^−1^ due to contributions from both labels in the outer strands. By contrast, the higher frequency band, associated with Leu13, has an intensity in **1W‐4**–**13**–**20** similar to that in both double‐labeled variants and in **1W‐13**, all of which have one label in the central strand. Thus, both labeled positions in **1W‐4**–**13**–**20** have equilibrium IR intensity patterns that reflect virtually independent spectral contributions to the overall band. Another impact of the introduction of multiple labels is a shift of the main β‐sheet band to higher wavenumbers (Table 1), which is largest for the triple‐labeled variant (up to 1642 cm^−1^). Disruption of the vibrational coupling in the β‐strands by isotopic substitution effectively shortens the coupled segments in the strand and results in less excitonic splitting.7a, 7b, 7d Thus, the higher intensity, lower frequency β‐strand component (1634 cm^−1^ in **1W**) is shifted to a higher wavenumber in the labeled variants due to disrupted coupling. For variants labeled in strand 1, this shift is ∼2–3 cm^−1^ more than that for those labeled in strand 3, which indicated removal of Leu4 has a stronger impact on β‐sheet coupling than that of Val20, and thus, reflects the higher degree of order in strand 1.

### Computed IR spectra

The isotope‐labeled three‐stranded structure does not show IR frequency shifts, as expected, from an ideal structure (Figure S2 in the Supporting Information), so we computed spectra for several structures determined by means of NMR (a representative one of which is shown in Figure S8 in the Supporting Information). In an ideal structure, coupling would result in splitting of the mode frequencies equally up and down from the mean of the single‐label positions and, for this geometry, the lower component would have most of the intensity. However, our experimental results for the double‐labeled variants show only smaller, asymmetric frequency shifts and reflect a simple summing of the single‐label results that indicate minimal coupling and suggest ideal simulations would be inadequate for explaining the observed spectra. Spectra were simulated by using peptides constructed with only Ala residues (except for two pairs of Aib−Gly turn residues), the torsions of which were constrained to values that corresponded to selected examples taken from the ensemble of best‐fit NMR‐determined structures. Spectral computations at only the BPW91/6‐31G** level, for the lowest energy NMR‐derived structure, gave the wrong relative frequency ordering for the labeled L4, L13, and V20 bands. Incorporating an implicit solvent correction with a polarizable continuum model (PCM) provided improvement, but addition of an empirical selective scaling of the FF for those C=O groups that pointed out to the solvent was required to obtain qualitatively improved relative frequencies.^12^ This correction empirically adjusts the single‐label frequencies to account for hydrogen bonding to water. If this were done quantum mechanically for explicit solvent, it would require both a very large calculation and unrealistically frozen or restricted water conformations. Even after correction, these calculations should be viewed as quite approximate, in that solvent and side‐chain effects are only empirically accommodated.

To test for sensitivity to conformational variation, we performed similar calculations for three different structures from the 10 best fits to the NMR‐determined constraints. The absolute frequencies for single‐labeled residues varied, but the relative frequency variation remained, with amide I modes of positions 4 and 20 being too high, with respect to that for position 13, even when using PCM corrections (data not shown). Only after alteration of the FF to account for the difference in solvation of edge versus central strands (Figure S8 in the Supporting Information) could we compute the qualitatively correct ordering for positions 4 and 13, compared with the experimentally observed frequencies (Table 1). For position 20, the frequency remained too high. By using these corrections, the mode character of the molecule, if double‐ or triple‐labeled, could be probed. The coupling constants for the labeled residue on the central strand (13) to the other strands are nonzero, but small, and coupling to the neighboring residues in the strand is just slightly stronger. The calculations do not show a stronger coupling for 4–13 relative to that of 13–20 (Figure S8 in the Supporting Information), in contrast to the experimental pattern.

### Site‐selective T‐jump dynamics

**Figure 3:**
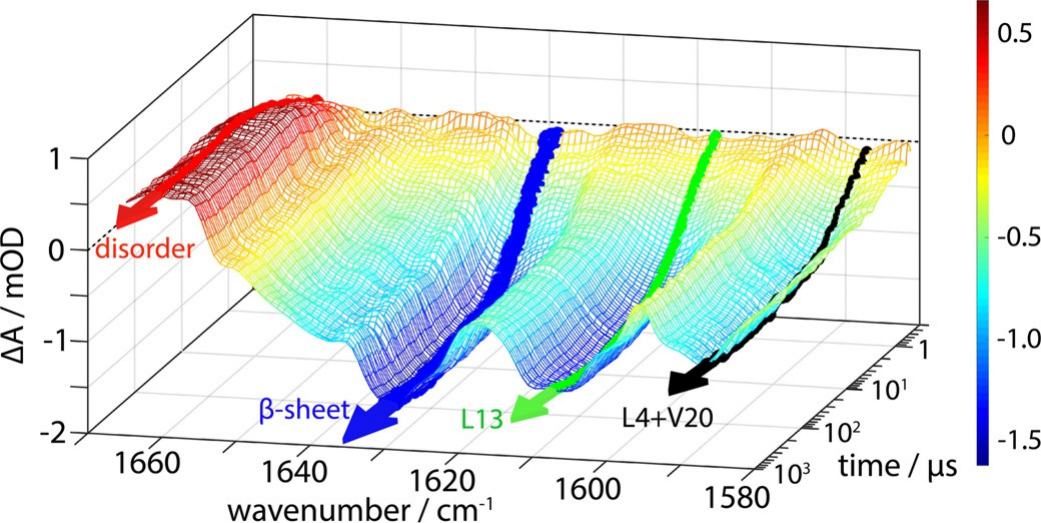
Example representation of the time‐dependent absorbance change of the amide I′ spectrum after the T‐jump for **1W‐4**–**13**–**20**. Data are shown over a time interval from 300 ns to 1 ms for a T‐jump from 9.3 to 15.3 °C, with transients recorded from 1581 to 1665 cm^−1^ in steps of 3 cm^−1^. The probe wavenumbers highlighted indicate contributions from the disordered structure (red), β‐sheet (blue), and the bands resulting from labeled residues at 1608 (green) and 1588 cm^−1^ (black). The arrows refer to fit lines and the color‐coding bar to absorbance changes.

Isotope labels can be used to visualize the dynamics and interactions of individual oscillators, while not altering the folding mechanism of the peptide. Figure 3 representatively shows, for the triple‐labeled peptide **1W‐4**–**13**–**20**, how site‐specific relaxation can be monitored by time variation of the entire amide I′ spectrum after a T‐jump. The disordered, β‐sheet, and isotope‐shifted bands give rise to four, resolved, dynamically detectable changes. Consistent with the equilibrium results above (Table 1), the changes in the ^13^C=O band associated with Leu13 (1608 cm^−1^) are clearly distinguished from those for Leu4 or Val20, which are spectrally unresolved (1588 cm^−1^), but have different, resolvable kinetic relaxations (see below). The change of absorbance after the T‐jump was remarkably strong for the labeled modes, in particular, for the Leu13 band at 1608 cm^−1^. Given the trends in Figure 3, it is sufficient to follow dynamics at only the peak frequencies to access their separate behaviors (see an example of single‐wavenumber transients in Figure S9 in the Supporting Information).

Relaxation dynamics of different parts of the peptide were determined at selected wavenumbers and for final temperatures over the range of ∼5–50 °C in the amide I' region based on their response in the difference spectra (Figure S5 in the Supporting Information). The loss of β‐sheet structure was monitored at ∼1629 cm^−1^ (Figure S10 a,b in the Supporting Information). Additionally, the rise of disordered structure was probed at ∼1662 cm^−1^ (Figure S11 a,b in the Supporting Information). The loss in intensity for the two ^13^C=O bands was probed at ∼1588 (Figure S12 a in the Supporting Information) and 1608 cm^−1^ (S12 b in the Supporting Information), respectively.

After correction for solvent contributions, the relaxation transients of the peptide were fit to a monoexponential function to derive rates. The observed time constants are less than 5 μs at 10 °C and ∼1 μs at 50 °C; these values are slightly slower than those for the related pG variants.8i A representative selection of relaxation times at 10 °C is given in Table 2. Unlike for a two‐state folding process, which should have uniform relaxation rates, the variation observed in Table 2 for selected wavenumbers indicates that different local dynamics are sampled, both in the shifted isotope bands and in the β‐sheet bands. These rate divergences are more evident at low temperatures, as shown in Figures S10–S12 in the Supporting Information.

**Table 2:** Relaxation times of differently labeled peptide variants at a final temperature of 10 °C.

Peptide	Relaxation time^[a]^ [μs]			
	Low‐frequency ^13^C=O (∼1588 cm^−1^)	High‐frequency ^13^C=O (∼1608 cm^−1^)	β‐Sheet (∼1629 cm^−1^)	Disordered (∼1662 cm^−1^)
**1W**	n.a.	n.a.	2.52(±0.43)	2.34(±0.46)
**1W‐4**	2.74(±0.63)	n.a.	2.03(±0.24)	2.66(±0.40)
**1W‐13**	n.a.	1.91(±0.29)	3.46(±0.36)	3.48(±0.48)
**1W‐20**	1.96(±0.42)	n.a.	2.34(±0.40)	3.25(±0.45)
**1W‐4**–**13**	1.67(±0.44)	2.86(±0.35)	3.22(±0.24)	3.14(±0.35)
**1W‐13**–**20**	1.30(±0.20)	3.22(±0.24)	3.20(±0.21)	3.49(±0.17)
**1W‐4**–**13**–**20**	1.66(±0.40)	3.20(±0.48)	2.89(±0.33)	3.42(±0.33)

[a] Values were obtained by fitting the temperature‐dependent kinetic data to the Arrhenius relationship to facilitate a comparison of relaxation times at one specific temperature for each band and variant; the error was determined by the regular residual as the mean of individual measurements over a temperature range of (10±3) °C.

**Figure 4:**
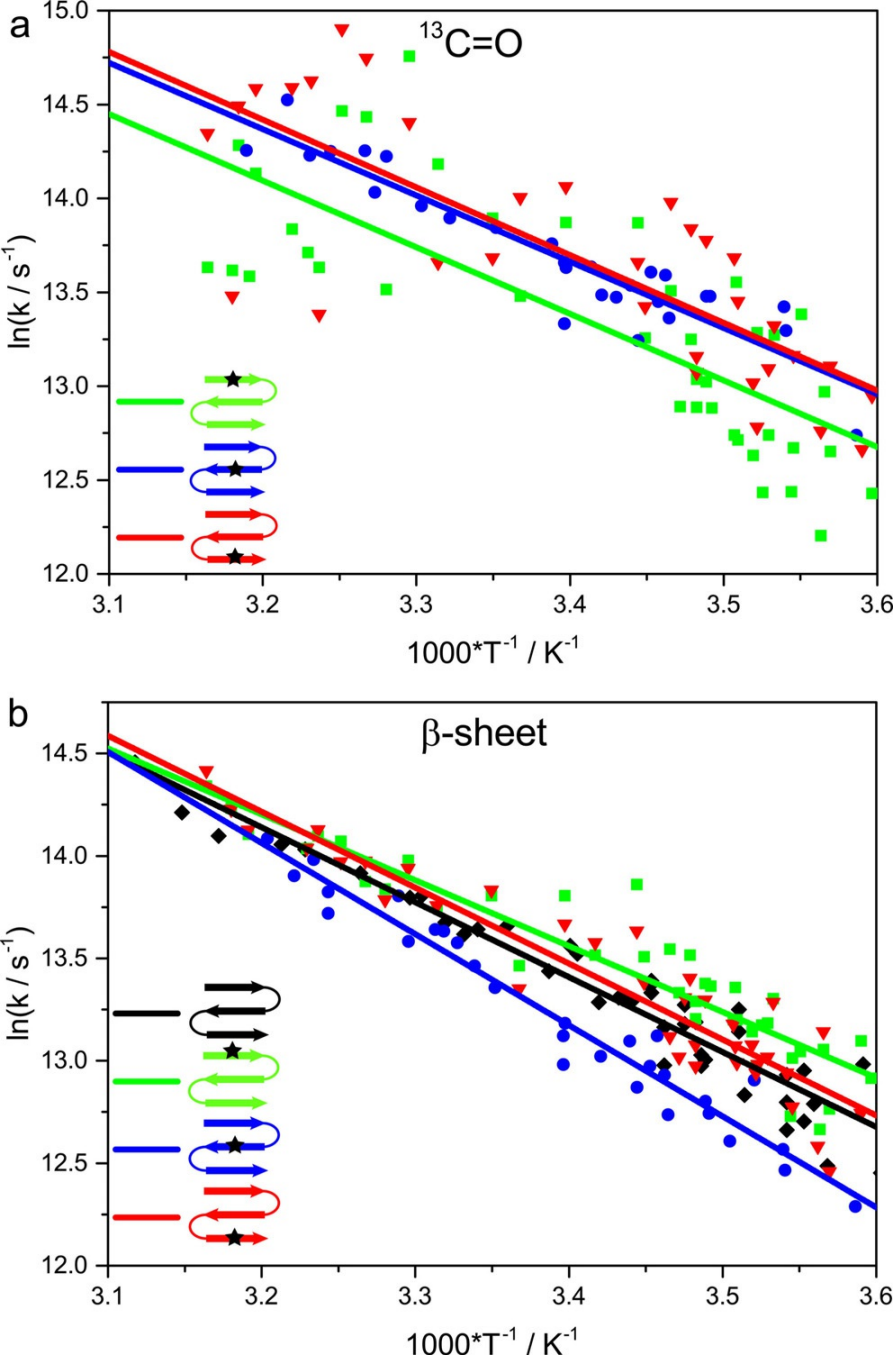
Relative relaxation behavior of the single‐labeled variants shown as Arrhenius plots. a) ^13^C=O modes probed at ∼1588 cm^−1^ for **1W‐4** (green squares) and **1W‐20** (blue circles) and ∼1608 cm^−1^ for **1W‐13** (red triangles), and b) β‐sheet contribution probed at ∼1629 cm^−1^, including **1W** (black diamonds). The lines represent fits to the Arrhenius equation to provide a qualitative description of the temperature dependence.

Considering the frequency‐shifted isotope modes (∼1588 and ∼1608 cm^−1^), the slowest relaxation time (*τ*=2.74 μs) is observed for the outer‐strand labeled carbonyl on **1W‐4**. The slow relaxation for the ^13^C=O band in the first strand is presumably due to stabilization of the first hairpin by the cross‐strand aromatic interaction.8g, 8i The labeled band for **1W‐13**, mostly describing the central strand, has a faster relaxation than that in **1W‐4**. The relaxation of the C‐terminal outer‐strand label for **1W‐20** is faster than that for **1W‐4** and similar to that for **1W‐13**. If attention is shifted to the overall β‐strand dynamics, measured at ∼1629 cm^−1^, the opposite trends occur, in that **1W‐13** has by far the slowest β‐strand change (*τ*=3.46 μs) and **1W‐4** and **1W‐20** are faster. Differences in relaxation time constants are small; however, trends are consistent not just for the 10 °C data shown in Table 2, but also over a wide temperature range, as observed from the Arrhenius profiles illustrated in Figure 4. Although the isotope bands of **1W‐4** and **1W‐20** both occur at ∼1588 cm^−1^, their best‐fit relaxation times differ for the entire temperature range, over which the band in **1W‐4** has slower dynamics than that in **1W‐20** (Figure 4 a). So, the less‐structured third strand has faster dynamics than the more ordered first strand, which fits expectations from the NMR‐determined structural disorder.

### Impact of labeling schemes on rate constants

**Figure 5:**
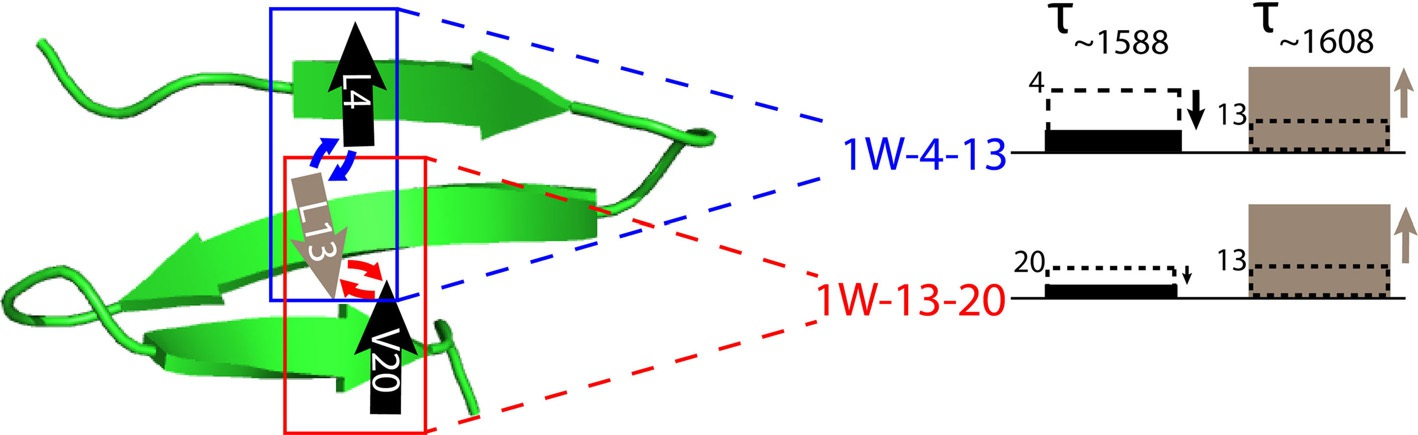
Impact of additional labels on the relaxation time constants measured for the labeled oscillators at ∼1588 and 1608 cm^−1^. The relaxation time constants for the single‐labeled residues (indicated by dashed boxes) are altered by interaction with an additional ^13^C=O. For the double‐labeled variants (indicated by solid boxes), smaller time constants are observed upon detection at ∼1588 cm^−1^ (black, Leu4 and Val20, respectively), whereas the opposite effect occurs at ∼1608 cm^−1^ (gray, Leu13).

**Figure 6:**
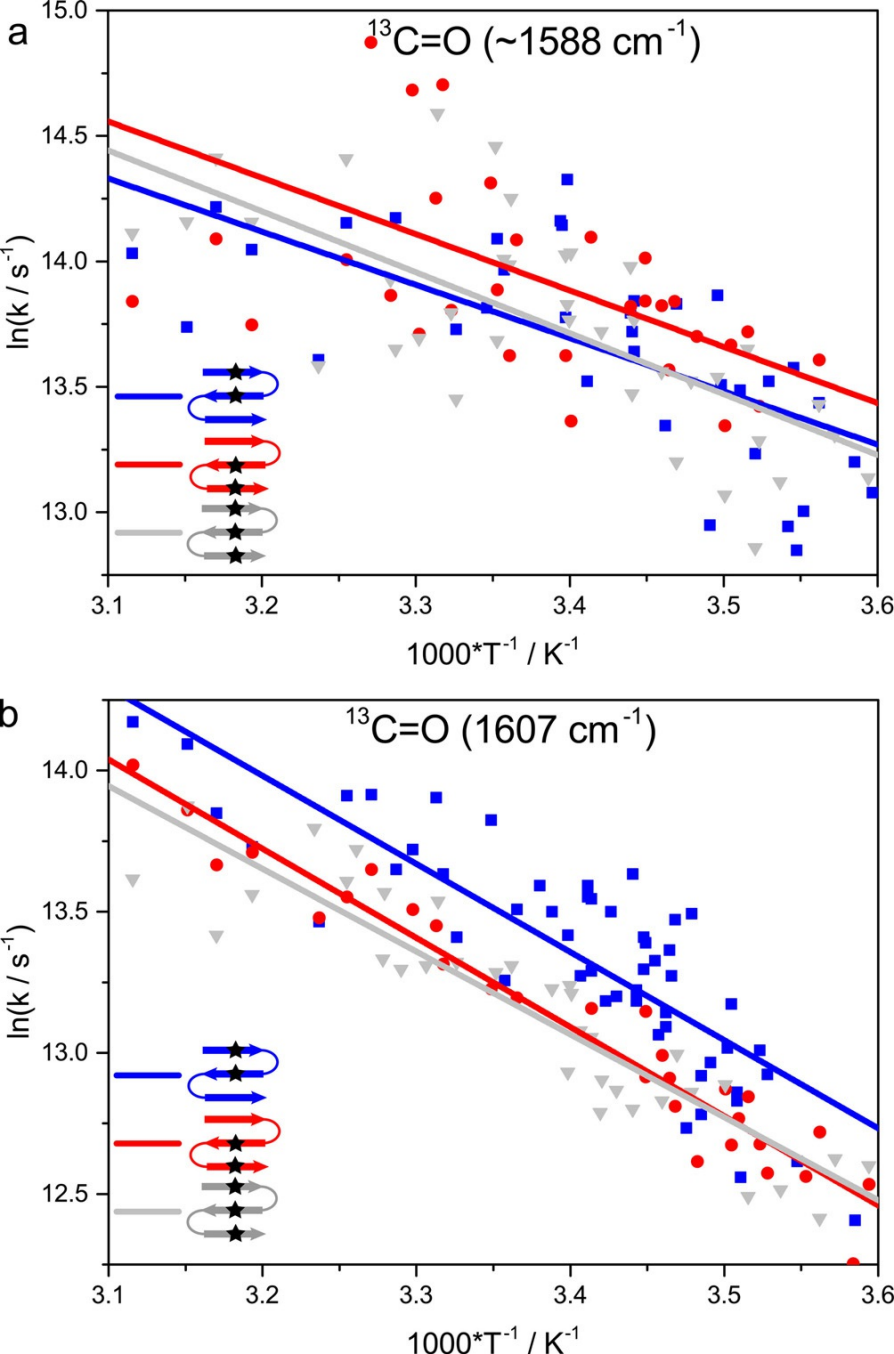
Relative relaxation behavior of the multiple labeled variants shown as Arrhenius plots. Isotope ^13^C=O modes were probed at ∼ a) 1588 and b) 1608 cm^−1^ for **1W‐4**–**13** (blue squares), **1W‐13**–**20** (red circles), and **1W‐4**–**13**–**20** (gray triangles). The lines represent fits to the Arrhenius equation to provide a qualitative description of the temperature dependence.

Our study clearly demonstrates that the observed rate constants probed at a selected wavenumber change depending on the labeling scheme. In the double‐labeled variants (Table 2), the pattern of Leu4 being slower than Val20 is reflected in the ^13^C=O band relaxation at ∼1588 cm^−1^ for **1W‐4**–**13** (*τ*=1.67 μs) being slower than that for **1W‐13**–**20** (*τ*=1.30 μs). Additionally, the incorporation of labels on different strands of the peptide impacts the relaxation kinetics for both sites, as indicated schematically in Figure 5. Substituting ^13^C=O to Leu13 decreases the time constants for both Leu4 and Val20. Inversely, the addition of ^13^C=O on either Leu4 or Val20 increases the time constants for Leu13. These experimentally observed changes reflect coupling and are represented in a temperature‐dependent manner in Figure 6, although cross‐strand interaction is less pronounced than it would be found for an ideal structure. Even weak coupling is revealed by a change in the time constant because added isotope substitutions contribute to the probed amide‐mode frequency. Thus, if an amide oscillator of another part of the peptide is isotopically labeled and couples to the probed oscillator, the resulting time constant will change accordingly, that is, if the coupling oscillator has a higher folding rate, the observed rate (*k*_obs_) gets higher and vice versa.

The triple‐labeled variant, **1W‐4**–**13**–**20**, containing contributions of both terminal strands has relaxations that partly reflect the dynamic behavior of the double‐labeled variants. At 1588 cm^−1^, relaxation is similar to that of **1W‐4**–**13**, and faster than those for **1W‐4** and **1W‐20**, whereas at 1608 cm^−1^ the relaxation is more like that of **1W‐13**–**20**, which is again slower than that for **1W‐13** (Table 2 and Figure 6). This might indicate that relaxation probed at 1588 cm^−1^ is dominated by strands 1–2, whereas relaxation probed at 1608 cm^−1^ reflects the coupling of strands 2–3. In summary, for both double‐ and triple‐label cases, the impact of coupling on the kinetics of differently labeled residues was detected with higher sensitivity than that possible by using equilibrium IR spectra.

### Alteration of kinetics by removal of distinct residue contributions

Bands associated with the unlabeled ^12^C=O residues, that is, the β‐sheet band at ∼1629 cm^−1^ and the disordered structure at ∼1663 cm^−1^, are affected when selected oscillators are shifted out of the main ^12^C=O band to lower frequencies by isotopic labeling. For example, removing the relatively slow dynamic contribution of Leu4 from the ^12^C‐β‐sheet band in **1W‐4** (Figure S10 in the Supporting Information) leads to faster relaxation at 1629 cm^−1^ (2.03 μs in comparison to 2.52 μs for the unlabeled peptide, at 10 °C). By contrast, removing Leu13 (which shows the fastest relaxation) on the central strand in **1W‐13** results in a significantly slower β‐sheet relaxation (3.46 μs, Table 2).

To some extent, the same pattern can be seen in the disordered band dynamics. For example, Val20 in **1W‐20** has a fast relaxation, yet relaxation for the disordered band at ∼1663 cm^−1^ in that variant is much slower than that for **1W**. This, in particular, applies to the frayed ends of the peptide. Dynamics for **1W‐21**, with the label one residue closer to the C‐terminal end, were also measured as a control, and yielded much slower kinetics for its labeled oscillator than that found for **1W‐20** (Figure S13 in the Supporting Information). Correspondingly, the kinetics for the disordered structure in **1W‐21** are faster than those for **1W** or **1W‐20**, whereas no significant difference was observed for its β‐sheet kinetics.

In the presence of multiple labels, several opposing effects originating from the individual label positions have to be considered. In general, the impact on relaxation rates of removing a residue from a sheet structure by isotopic labeling is roughly additive, which means that removing a fast relaxing residue slows the rate for what remains unlabeled. The opposite change occurs, that is, increasing the rate for the unlabeled component, if a slow relaxing residue is removed. This applies to relaxation times observed at both ∼1629 and 1663 cm^−1^, for the β‐strand and disordered components, respectively.

### Local dynamics, stabilities, and relaxation rates

We have made a series of isotopically labeled three‐stranded hairpins and showed measurably different dynamics for selected positions within the strands. It seems clear from these variable rates that this model β‐sheet peptide is a multistate folder. Investigation of the structure and spectral consequences can provide some insight into the local dynamics, as our data has exposed.

With regard to the single‐labeled variants, substitution on the Leu4 residue (**1W‐4**) yields the slowest relaxation rates. Recalling that the equilibrium thermal transition has a *T*_m_ of ∼73 °C, at this point *k*_f_=*k*_u_, if we restrict the description to a simple two‐state analogy for the overall folding and unfolding rate constants. All of our data sample relaxation after heating, but well below the equilibrium transition, that is, we operate under conditions in which the folded fraction, *f*_f_, is greater than that of the unfolded fraction, *f*_u_. Consequently, because *k*_f_/*k*_u_∼*f*_f_/*f*_u_>1 for *T*<*T*_m_, *k*_f_>*k*_u_ and the observed relaxation rate constant, *k*_obs_∼*k*_f_+*k*_u_, must be dominated by *k*_f_. The stability of the first strand or first hairpin is enhanced by the Trp−Tyr cross‐stranded aromatic contact, which, as we have previously shown, leads to slower contributions to the global relaxation kinetics.8g, 8i Here, we see that slower *k*_obs_ is characteristic of the local dynamics of Leu4 as well, and presumably would mean *k*_f_ is slower. By contrast, the *k*_obs_ values for Leu13 and Val20 are similar. These two residues are hydrogen bonded in a small ring, which is characteristic of the antiparallel β‐sheet structure.7b, 7c, 13 Since we detect dynamics by a change in absorbance at a selected wavenumber, as the strands separate in the unfolding/folding process, the absorbance for both will change, leading to their similar relaxation rates.

For double‐labeled samples, we can detect and monitor the dynamics of each labeled residue separately, in contrast to our initial expectations. Consequently, the effects of coupling on the IR spectrum, intensity and frequency distribution, are reduced in such nondegenerate oscillator systems. However, the labeled residues impact each other in interesting ways that are enhanced in the dynamics. Monitoring the Leu13 dynamics (1608 cm^−1^, Figure 6 b) under the influence of an additional label on Leu4 or Val20, we see a decisive slowing of relaxation for **1W‐4**–**13** and **1W‐13**–**20**, respectively. If we consider Leu13 to be in the most regularly folded part of the peptide, then *k*_f_ for it should be fastest, that is, most favored to form. Adding Leu4 to this in **1W‐4**–**13** can only slow *k*_f_, since Leu4 is in a less folded segment, but this impact on the Leu13 band must be due to coupling to Leu4. If we regard the third strand as even more disordered, as observed in our MD and NMR results, then the additional slowing of *k*_f_ for Leu13 in **1W‐13**–**20** can be understood. Alternatively, if we consider relaxation at 1588 cm^−1^, that is, for the labeled Leu4 or Val20 bands, and add Leu13, as is the case in **1W‐4**–**13** or **1W‐13**–**20**, respectively, then we are adding a more structured part of the molecule to the detectable relaxation process, and thus, *k*_f_ increases through coupling again, as observed. These trends are all observed in Table 2 for relaxation kinetics at 10 °C, but are also apparent in the global trends over the range of 5–50 °C, as shown in Figures 4 and 6. Viewing the data in the Arrhenius‐style format, of log *k*_obs_ versus 1/*T*, helps to visualize these comparisons a bit more easily than that in terms of relaxation time constants versus *T*, but the relative differences are also evident in these alternate‐style plots (Figures S10–12 in the Supporting Information).

## Conclusion

Spectral effects of isotopic labeling are highly sensitive to molecular structure and dynamics. If multiple vibrationally coupled sites are labeled, there is potential for a more detailed structural interpretation of the data that derives from their through‐space and through‐bond couplings. Our study reveals that vibrational coupling is more sensitively probed by measuring site‐selective kinetics than by analyses of equilibrium IR spectra. Even if the oscillators are not degenerate, resulting in conditions for which couplings can be difficult to determine from frequency shifts in the equilibrium spectra, dynamic studies offer a novel way to identify weak couplings. We observed that relaxation dynamics detected at single wavenumbers depended significantly on the contributing coupled oscillators. Differences in time constants reflect different couplings obtained with varying labeling schemes. It is important to note that the folding mechanism of the peptide is not affected by any isotopic substitutions, so that the incorporation of multiple ^13^C=O labels can provide a sensitive, perturbation‐free means of probing local conformational dynamics. The enhancement in sensitivity to coupling observed herein in the analysis of the dynamics is a new development and can open up the investigation of larger systems, in which single isotopic labels might be too dilute to generate measurable effects in equilibrium IR.

## Conflict of interest

The authors declare no conflict of interest.

## Supporting information

As a service to our authors and readers, this journal provides supporting information supplied by the authors. Such materials are peer reviewed and may be re‐organized for online delivery, but are not copy‐edited or typeset. Technical support issues arising from supporting information (other than missing files) should be addressed to the authors.

Supplementary
