# Supplementary material for: Enhanced Sensitivity to Local Dynamics in Peptides by Use of Temperature‐Jump IR Spectroscopy and Isotope Labeling
Source: Chemistry. 2020 Feb 4;26(16):3524–34. doi: 10.1002/chem.201904497 (PMC7155074; doi:10.1002/chem.201904497)
Supplement: Supplementary file 1 — Supplementary [file CHEM-26-3524-s001.pdf]

CHEMISTRY
A European Journal

Supporting Information

Enhanced Sensitivity to Local Dynamics in Peptides by Use of Temperature-Jump IR Spectroscopy and Isotope Labeling

David Scheerer,[a] Heng Chi,[b, c] Dan McElheny,[b] Timothy A. Keiderling,*[b] and Karin Hauser*[a]

chem_201904497_sm_miscellaneous_information.pdf

Table S1. NMR structure solution data

Total unambiguous distance restraints	411
intraresidue (i,i)	114
sequential (i, i+1)	119
medium range (2 ≤ |i-j| ≤ 4)	62
long range (|i-j| > 4)	116
intermolecular	0
Total dihedral angle restraints	11
Phi	11
Psi	0
Chi1	0
Backbone atoms - RMSD from mean	0.517
All heavy atoms - RMSD from mean	0.918
Violation analysis	
Max dihedral angle violation (deg)	0.53
Max distance violation (A)	2.16
Mean distance violation - distance constraint (A)	0.635 +/- 0.314
Mean dihedral angle violation - dihedral constraints	0.520 +/- 2.649
Deviation from ideal bond lengths – (mean of 10)	0.00979 +/- 0.00012
Deviation from ideal bond angles – (mean of 10)	1.73540 +/- 0.04025

Procheck (pdb core allowed generous disallowed)

pdb	core	allowed	generous	disallowed
1	85.0	15.0	0.0	0.0
2	80.0	20.0	0.0	0.0
3	85.0	15.0	0.0	0.0
4	90.0	10.0	0.0	0.0
5	85.0	15.0	0.0	0.0
6	90.0	10.0	0.0	0.0
7	80.0	20.0	0.0	0.0
8	95.0	5.0	0.0	0.0
9	80.0	20.0	0.0	0.0
10	90.0	10.0	0.0	0.0

Energies

mean AMBER energy (kcal mol^{-1})	-710.61
mean restraint energy (kcal mol^{-1})	12.24

TOP 10 Energies (kcal/mol^{-1})

-717.5, -714.1, -713.7, -713.1, -710.9, -710.9, -710.7, -710.4, -709.6, -709.5

Computational details.

Density functional calculations (DFT). DFT simulations of the force fields (FF) and IR intensities were carried out on a 23-residue sequence converted to all Ala, except for the two pairs of Aib–Gly turn residues. This reduced the calculation size but maintained the full peptide length and geometry. Conformations were constrained to sets of (ϕ,ψ) torsions for selected structures determined as the best fits to the NMR data for the closely related, similarly structured peptide, SVKIWTS-BG-KTYTEV-BG-TKTLQE-NH_2. Computations were realized using Gaussian16[1] on a multi-processor Linux system at the BPW91/6-31G**/PCM level with implicit water simulation (PCM) as it was done previously for the related **pG2** sequence with ^{D}Pro-Gly turns.[2] These relatively large calculations enabled an analysis of the vibrational modes and their spectral properties without the need for fragmentation or transfer of parameters,[3] however, even when residues are restricted to just Ala, their size precludes full computation of explicit effects of side-chains or solvent. Spectra for isotopically labeled variants were simulated by incorporating the appropriate masses and re-diagonalizing the FFs using programs provided by Prof. Petr Bouř, Czech Academy of Science, Prague.[4] The peptides, particularly for residues near the N- and C-termini have dynamic fluctuations, so dependence on a static spectral simulation is limited in its ability to reflect experimental results. Consequently, we performed a few DFT-based simulations with different NMR-determined low-energy geometries to estimate spectral sensitivity to structural variations (see a representative example in Fig S8). For comparison we also computed spectra for an ideal, fully minimized three-strand structure, as shown in Figure S2.

Molecular dynamics simulations. The lowest energy NMR structure of SVKIWTS-BG-KTYTEV-BG-TKTLQE-NH_2 was solvated into a rectangular box of TIP3P water molecules (~3500) and subjected to unrestrained molecular dynamics simulations for 200 ns. Calculations closely followed those previously reported for **pG2** [2] and again used the Amber force field FF14SB on a workstation optimized for the Amber 14 package.[5] The entire system was initially energy minimized with 5000 steps of steepest descent followed by 5000 steps of conjugate gradient minimization, and then seven stages of heating and equilibration, with ramping down restraints at each stage as done previously.[2] All covalent bonds involving hydrogen atoms were restrained with the SHAKE algorithm.[6] Electrostatic interactions were treated with the particle mesh Ewald (PME) summation having a cutoff of 12 Å. An unrestrained NPT MD was then allowed to run for an additional 200 ns from which snapshots of the trajectories were stored every 10 ps. CPPTRAJ[7] was used to analyze the trajectories for information such as torsional angles and distances between atoms.

Figure S2. DFT simulations of vibrational spectra for a **fully minimized**, unrestrained three-stranded peptide (top). ^{13}C-isotopic substitution was performed on the same amide C=O´s as used for the **1W** peptides, namely positions **4** (a), **13** (b) and **20** (c). Mixing of the modes was simulated by the introduction of multiple labels, as **4-13** (d), **13-20** (e) and **4-13-20** (f). Spectra are simulated after deuteration of all exchangeable Hs. The FF was not modified to reflect the effects of explicit water solvation of outer strands, therefore oscillators not H-bonded to other strands (like Leu4) develop frequencies that are too high. The spectral effects of different solvent interactions and a realistic conformation are shown for an example in Figure S8. The band width was chosen to be 10 cm^{-1} full width at half maximum. The shift of intensity to the lowest frequency mode (**13-20**, **4-13-20**) is indicative of strong coupling effects. **4-13** shows less coupling since the ^{13}C=O oscillator frequencies are quite different.

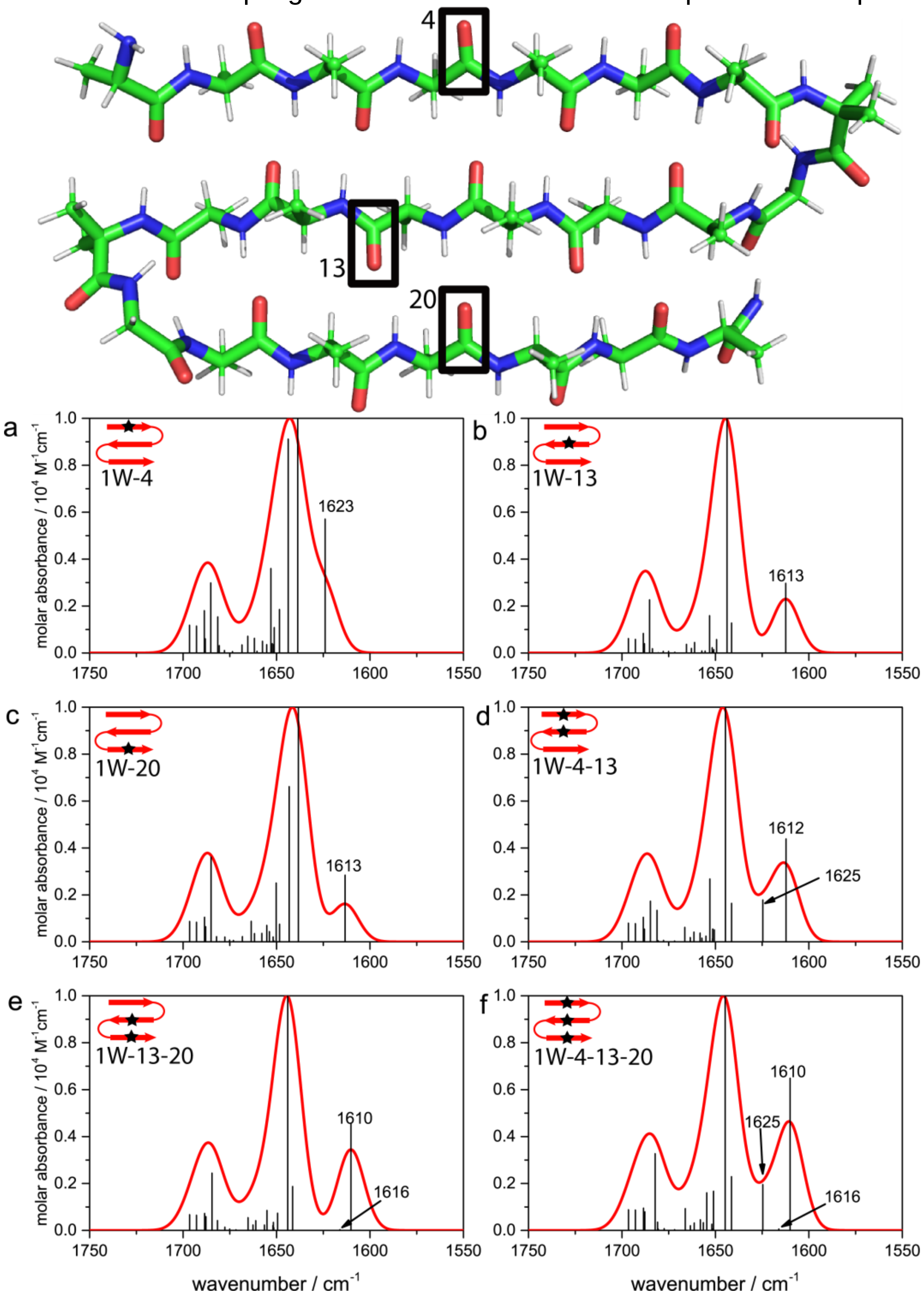

Table S3. Comparison of the ϕ,ψ variation in **a)** turn 1 and **b)** turn 2. For both Aib and Gly, ϕ and ψ angles are given as an average of the angles in the ten best NMR-fit structures and for computed structures derived from the MD trajectories (sampled every 100 ps along the 200 ns long trajectories).

a)

	Aib8		Gly9	
	ϕ	ψ	ϕ	ψ
NMR	52.7(±0.7)	32.8(±2.1)	71.0(±5.4)	19.0(±14.8)
MD	54.7(±7.8)	30.6(±14.8)	81.0(±23.3)	-4.3(±27.2)

b)

	Aib16		Gly17	
	ϕ	ψ	ϕ	ψ
NMR	52.7(±0.7)	32.4(±1.8)	76.6(±6.8)	6.8(±19.1)
MD	55.5(±7.3)	28.8(±11.2)	78.5(±19.4)	9.9(±26.6)

Table S4. Comparison of the H-bond distances in Å between strands **a)** 1–2 and **b)** strands 2–3 in the ten best NMR-fit structures and computed structures from the MD trajectories (sampled every 100 ps along the 200 ns long trajectories). The pairs furthest from the turn (3N-14C and 11N–22C, respectively) have larger separations, indicating fraying of the ends.

a)

	Strand 1–2			
	3N–14C	3C–14N	5N–12C	5C–12N
NMR	4.58(±0.53)	3.96(±0.10)	4.04(±0.05)	3.95(±0.05)
MD	6.67(±0.83)	5.12(±0.58)	4.14(±0.15)	4.03(±0.12)

b)

	Strand 2–3			
	11N–22C	11C–22N	13N–20C	13C–20N
NMR	5.43(±0.98)	3.86(±0.07)	4.10(±0.01)	4.06(±0.05)
MD	5.53(±0.54)	3.87(±0.21)	4.10(±0.13)	4.05(±0.15)

Equilibrium IR. For IR measurements, peptide samples were dissolved in D_2O at ~10 mg/mL at acidic pH (after lyophilization from DCl for removal of TFA and H/D exchange) and were placed in a homemade demountable cell consisting of CaF_2 windows separated by a Teflon spacer (100 µm optical pathlength). IR spectra were measured with a Bruker Equinox 55 FTIR spectrometer equipped with a mercury cadmium telluride detector. For each FTIR spectrum, 128 scans with a spectral resolution of 4 cm^{-1} were averaged at each temperature. Temperature dependent IR spectra were measured in the temperature range of 5–95 °C in steps of $\Delta T = 5$ °C. The temperature of the sample holder was controlled by flow from a water bath (Lauda Ecoline E300, Germany), with the sample cell temperature recorded by a Pt100 sensor. A self-written script running in MATLAB (MathWorks, USA) was used for subtraction of the solvent absorption from the peptide spectra. The respective D_2O and HDO contents of the peptide spectra were determined at each temperature by the use of temperature-dependent reference spectra of D_2O and D_2O/H_2O mixtures and the characteristic bands at 3840 cm^{-1} (D_2O) and 3400 cm^{-1} (HDO). This allows for the correct scaling and subtraction of the solvent contribution. In addition, in regions of no or minimal peptide absorbance (1850-1800 cm^{-1} as well as 1550-1530 cm^{-1}) flat baselines are created. Positions of band maxima were determined by use of the 2nd derivatives of the absorption spectra.

Figure S5. Comparison of the temperature dependent IR absorption of the amide I’ band for all peptide variants in D_2O solution between 5 and 95 °C **a) 1W**, **b) 1W-4**, **c) 1W-13**, **d) 1W-20**, **e) 1W-4-13**, **f) 1W-13-20** and **g) 1W-4-13-20**. Difference spectra (right column) were obtained by using the 5 °C spectrum as a reference.

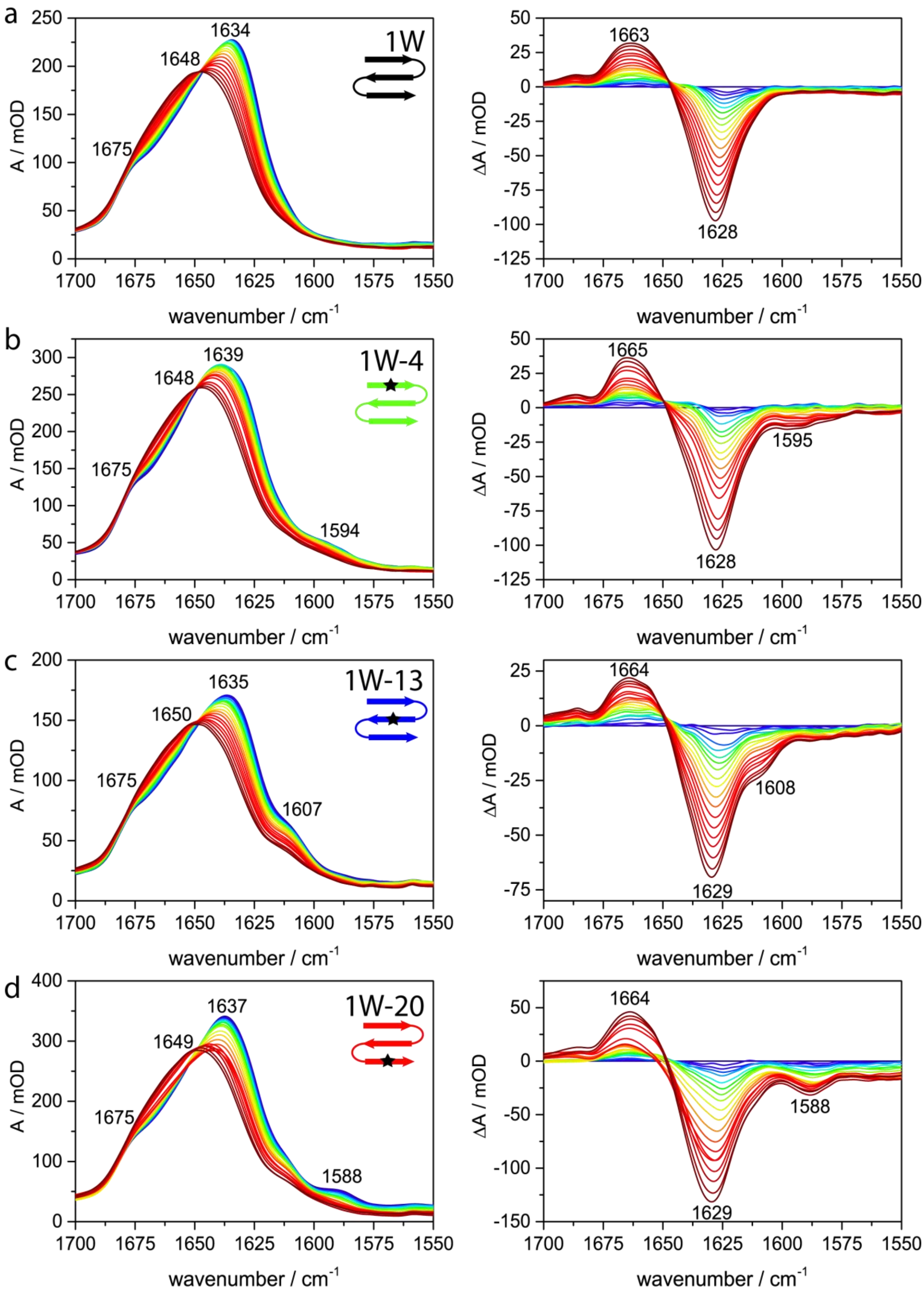

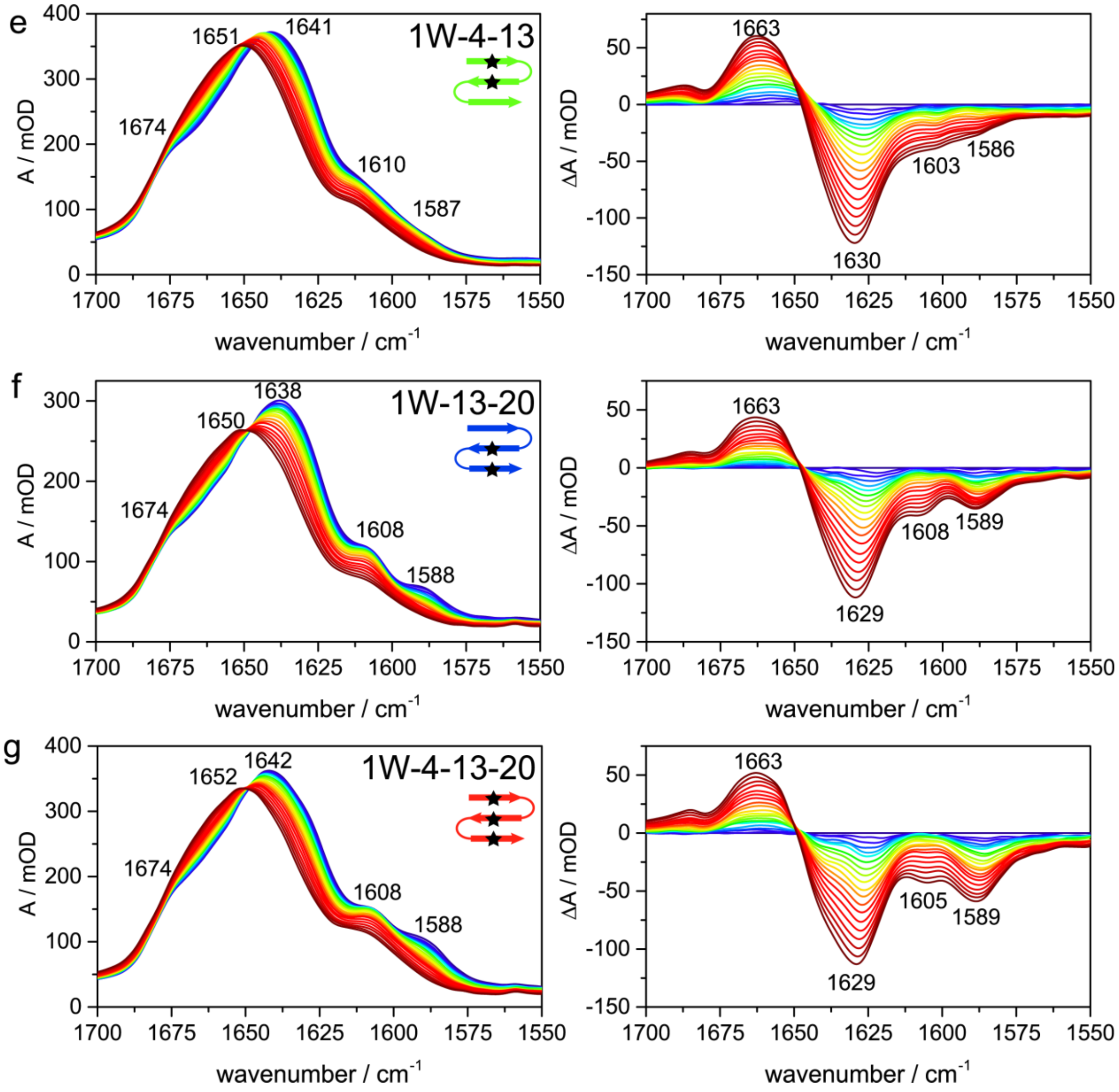

e
1W-4-13
1651
1641
1674
1610
1587
1663
1630
1603
1586
f
1W-13-20
1638
1650
1674
1608
1588
1663
1629
1608
1589
g
1W-4-13-20
1642
1652
1674
1608
1588
1663
1629
1605
1589
A / mOD
ΔA / mOD
wavenumber / cm-1

Figure S6. IR monitored thermal transition shown for **1W** as an example. The transition was derived from the variation of the 2nd component of a singular value decomposition (SVD) of the set of temperature dependent IR spectra analyzed over only the amide I´ region. SVD of each set of temperature dependent spectra, examples of which are shown in Figure S5, was obtained using MATLAB software. The very broad transitions measured for three different replicate **1W** samples (blue triangles, black squares and green circles) were globally fit (red line) to a sigmoidal transition function with flat baselines as follows:

$$y = A_2 + (A_1 - A_2)/[1 + exp((T - T_m)/\mathrm{d}T)]$$

where A_1 is the initial value, A_2 the final value and $\mathrm{d}T$ the slope factor. A value of T_m = 82(±3) °C was obtained, however we were concerned about its accuracy, due to the broad transition and our assumption of flat baselines. While fitting a flat baseline might be a reasonable approximation at low temperatures, this is not the case at high temperatures.

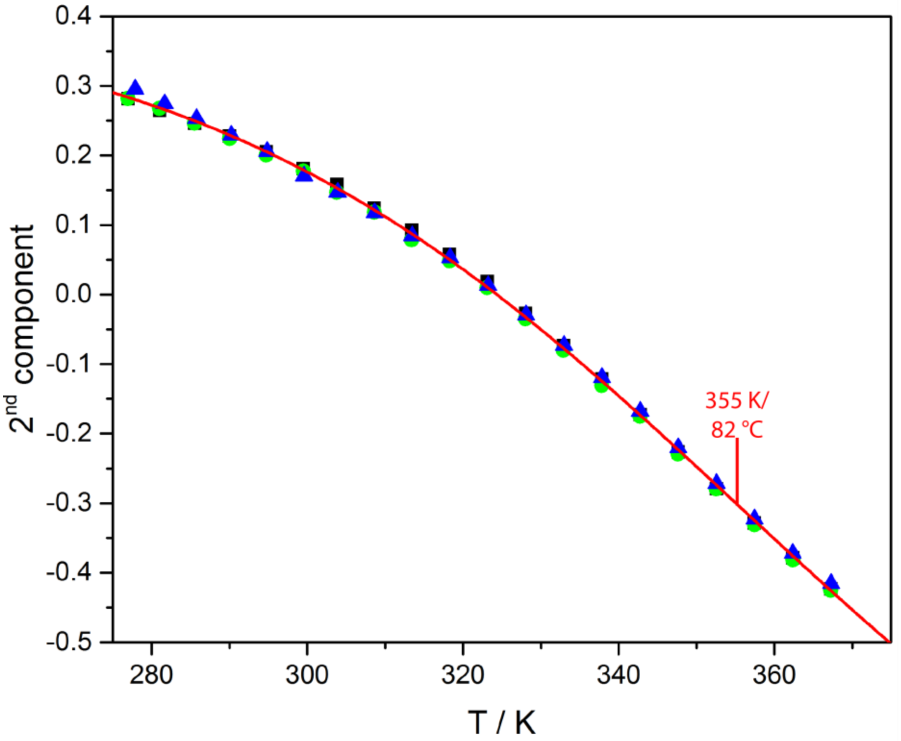

Therefore, data were numerically differentiated with respect to the temperature.[8] The first derivative was fit to an apparent two-state equilibrium model:

$$\frac{\mathrm{d}(signal)}{\mathrm{d}T} = Af(1-f)T^2$$

where d*(signal)/*d*T* is the algebraic derivative of the 2nd component, *A* is a scaling factor and *f* is the fraction of denatured peptide. For a two-state transition, *f* is related to the melting temperature (T_m) and van't Hoff enthalpy (ΔH_{vm}) by the equilibrium constant (K) for the unimolecular reaction between the denatured and folded state:

$$f = \frac{K}{K+1}$$

$$K = exp\left[\frac{\Delta H_{\mathrm{vm}}}{R}\left(\frac{1}{T_m} - \frac{1}{T}\right)\right]$$

To test the sensitivity of our revised fit and determination of T_m, we added y-axis shifts to simulate errors in pre- and post-transitional baselines of the 2nd component.[8] The T_m of 73(±1) °C was mostly unaffected by y-axis translation. For a y-translation of ±0.001 arbitrary units, T_m changed by ±1 °C. In contrast, ΔH_{vm} (-50(±14) kJ/mol) shows a stronger variation (±28 %) for the same baseline shift. Consequently, we will limit ourselves here to using the equilibrium transition analysis to indicate just the transition temperature.

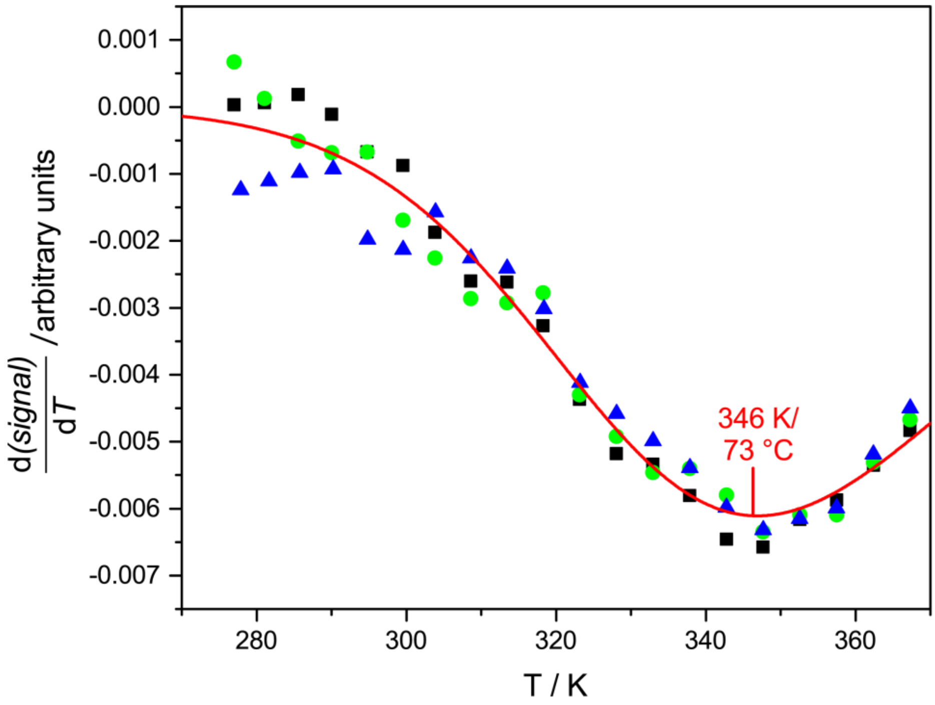

Figure S7. IR absorption spectra of the amide I' band of the single labeled variants, which can also be used to provide added data for correcting the force field. In each plot the spectrum of the unlabeled variant **1W** (black) is provided for comparison. **a)** Schematic representation indicating the label positions. **b)** Amide I' spectra for variants labeled on the Gly residues in the turns. **1W-9** (red) has a relatively intense isotope shifted band, while that for **1W-17** (blue) is broad. **c)** Amide I' spectrum for **1W-15** (green) also has a relatively intense isotope shifted band, while that of **1W-21** (purple) is broader and lies to higher wavenumbers, presumably due to its location close to the disordered C-terminal end.

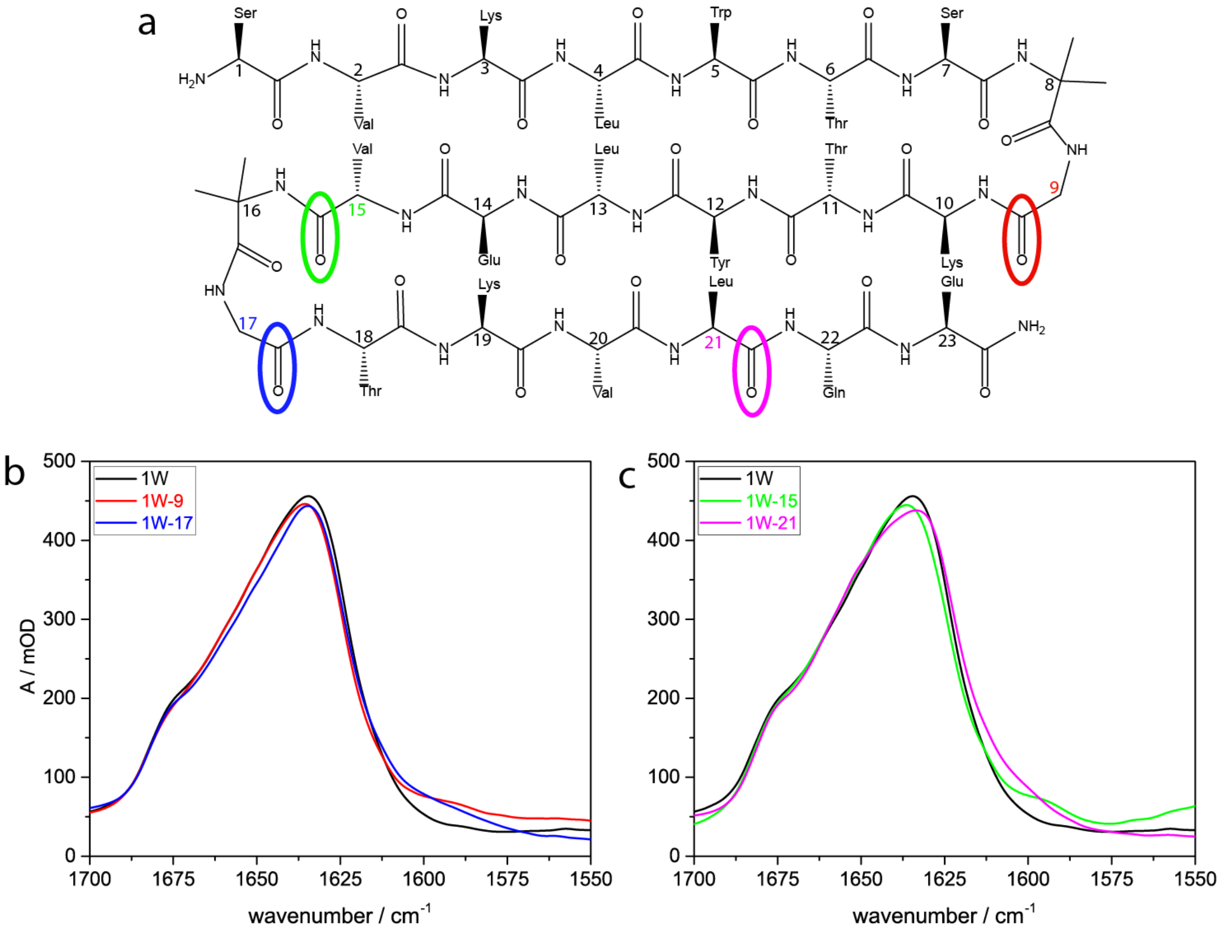

Band positions of ^{13}C-labeled and β-sheet modes in cm^{-1} from the IR absorption spectra:

peptide	^{13}C=O	β-sheet
**1W**	-	1634
**1W-9**	1591	1635
**1W-17**	1592	1634
**1W-15**	1594	1636
**1W-21**	1600	1633

Figure S8. Amide I' absorbance spectra simulated using DFT level calculations of an all alanine peptide constrained to torsional angles corresponding to one **representative NMR solution structure** (shown at the top), for which the N- and C-terminal ends were relatively well aligned with the center strand, while still forming a highly twisted sheet. The amide C=O´s marked with black boxes were chosen for isotopic labeling, namely to simulate spectra for **a) 1W-4**, **b) 1W-13**, **c) 1W-20**, **d) 1W-4-13**, **e) 1W-13-20** and **f) 1W-4-13-20.** Spectra are simulated after deuteration of all exchangeable Hs for these variants. The component bands were chosen to be 10 cm^{-1} full width at half maximum and summed to represent the overall amide I' envelop. To better account for the spectral impact of different solvent interactions for the outer- and center-strands, modifications of the FF were introduced. For all the edge C=Os, which in the NMR structures point out at the solvent, the diagonal FF was scaled using an empirically adjusted scaling factor,[2] and the resulting amide I frequency distributions were compared to experimental IR results to obtain just qualitative overall fits.

The absolute frequencies computed for single labeled residues varied for different structures obtained from the NMR best-fit set, however the relative trends for substitutions on various positions remained. As compared to experimentally observed frequencies (Table 1) the qualitatively correct ordering was obtained for positions 4 and 13. For position 20, the computed frequencies were too high, but still could be used to determine the molecule´s mode character when double or triple labeled. Due to the non-degeneracy of the different single-labeled modes, weaker coupling was found for the multiply labeled variants than seen experimentally or for the ideal case. This can be seen by contrasting these results (Fig. S8) with those for a more ideal structure (Fig. S2).

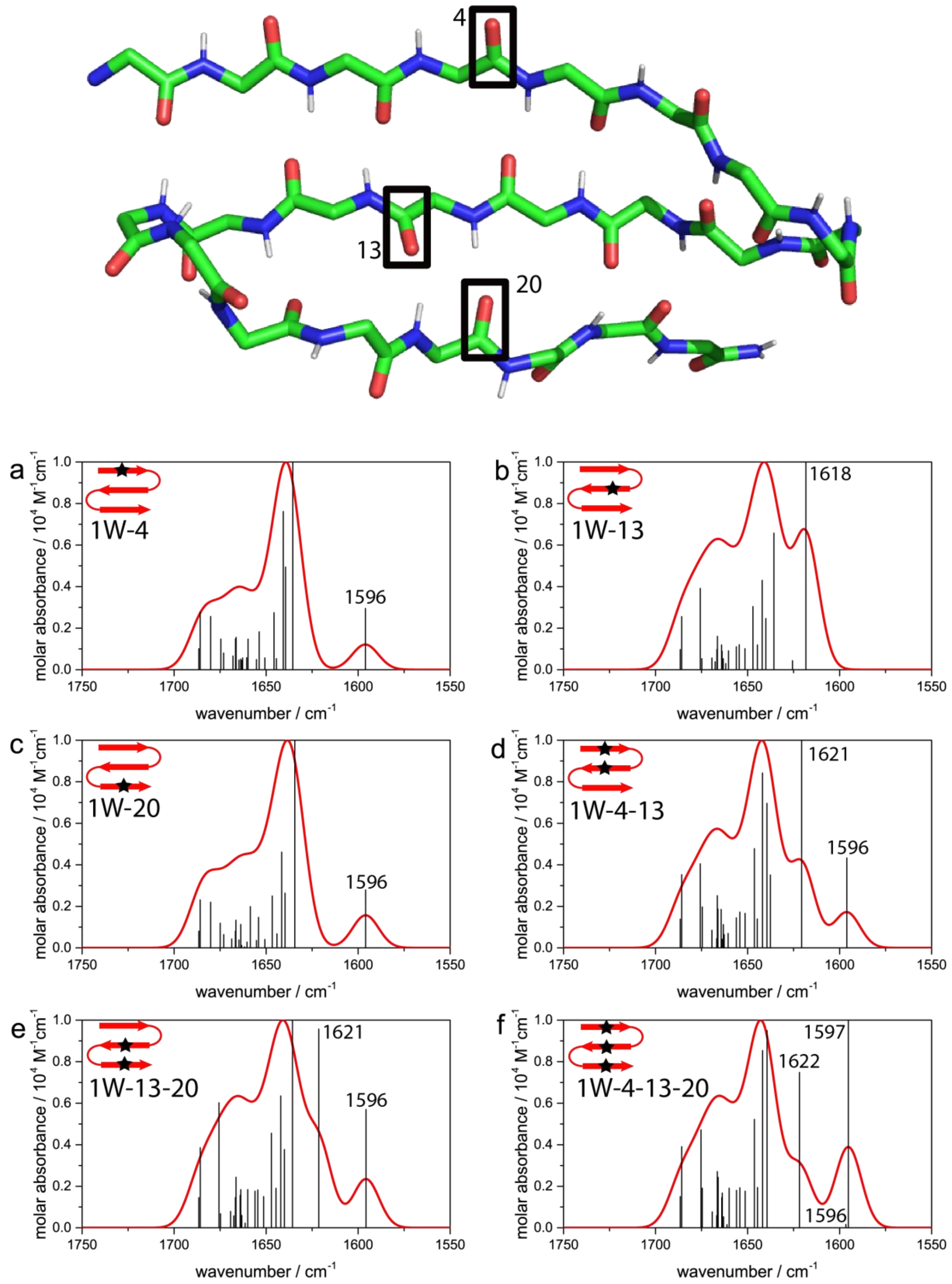

4
13
20
a
1W-4
1596
b
1W-13
1618
c
1W-20
1596
d
1W-4-13
1621
1596
e
1W-13-20
1621
1596
f
1W-4-13-20
1597
1622
1596
molar absorbance / 10^4 M^{-1}cm^{-1}
wavenumber / cm^{-1}

Figure S9: Example of transient relaxation after a laser-excited T-jump from 11.0 to 17.5 °C for different probe wavenumbers, here shown for **1W-4-13-20**. The strongest change in absorbance can be observed for the β-sheet band at 1629 cm^{-1} (blue triangles). For the disordered structure at 1663 cm^{-1} (red diamonds) an increase in absorbance can be observed. The two bands monitored at 1608 cm^{-1} (green circles) and 1588 cm^{-1} (black squares), which result from isotopic labeling, show less but still significant change. Transient data below 300 ns (faded points) was perturbed and much less reproducible, and thus was not included in the single-exponential fits (solid lines). Relaxation kinetics for ~1000 transients were averaged. The reversibility of the folding and unfolding transition was confirmed by acquiring FTIR spectra subsequent to the T-jump experiments. The samples studied are fully relaxed back to the initial temperature after ~100 ms, thus excitation at 5 Hz repetition rate ensures that the T-jump is applied at the same initial conditions.

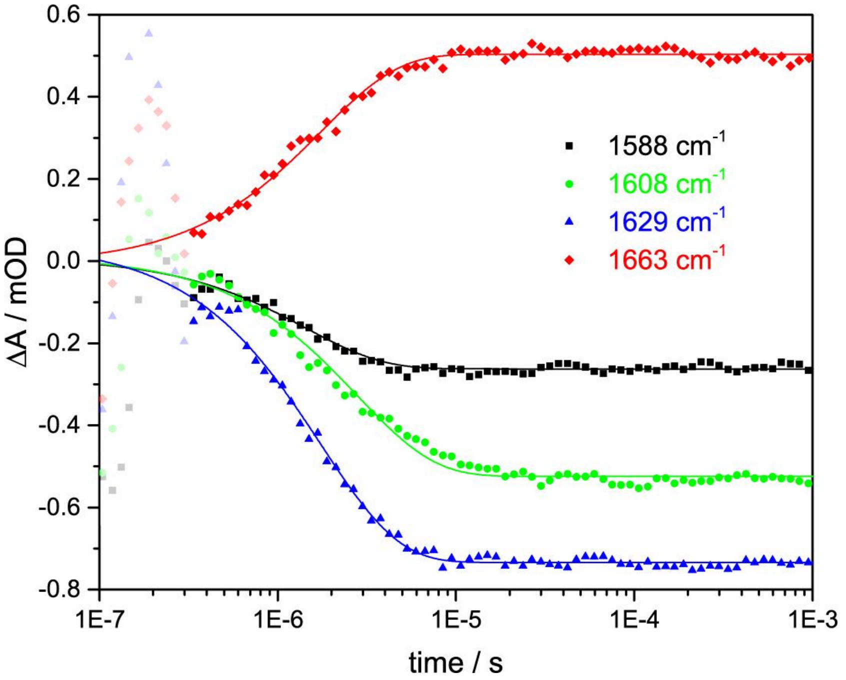

Figure S10. Comparison of the β-strand relaxation times at 1629 cm^{-1} for **a)** single labeled **1W**-variants **1W-4** (green squares), **1W-13** (blue diamonds) and **1W-20** (red circles) in comparison to the unlabeled **1W** (black triangles), and **b)** multiple labeled **1W**-variants **1W-4-13** (green squares), **1W-13-20** (blue diamonds), **1W-4-13-20** (red circles) in comparison to the unlabeled **1W** (black triangles). The lines represent fits to the Arrhenius equation as a qualitative description of the temperature dependence of the relaxation times.

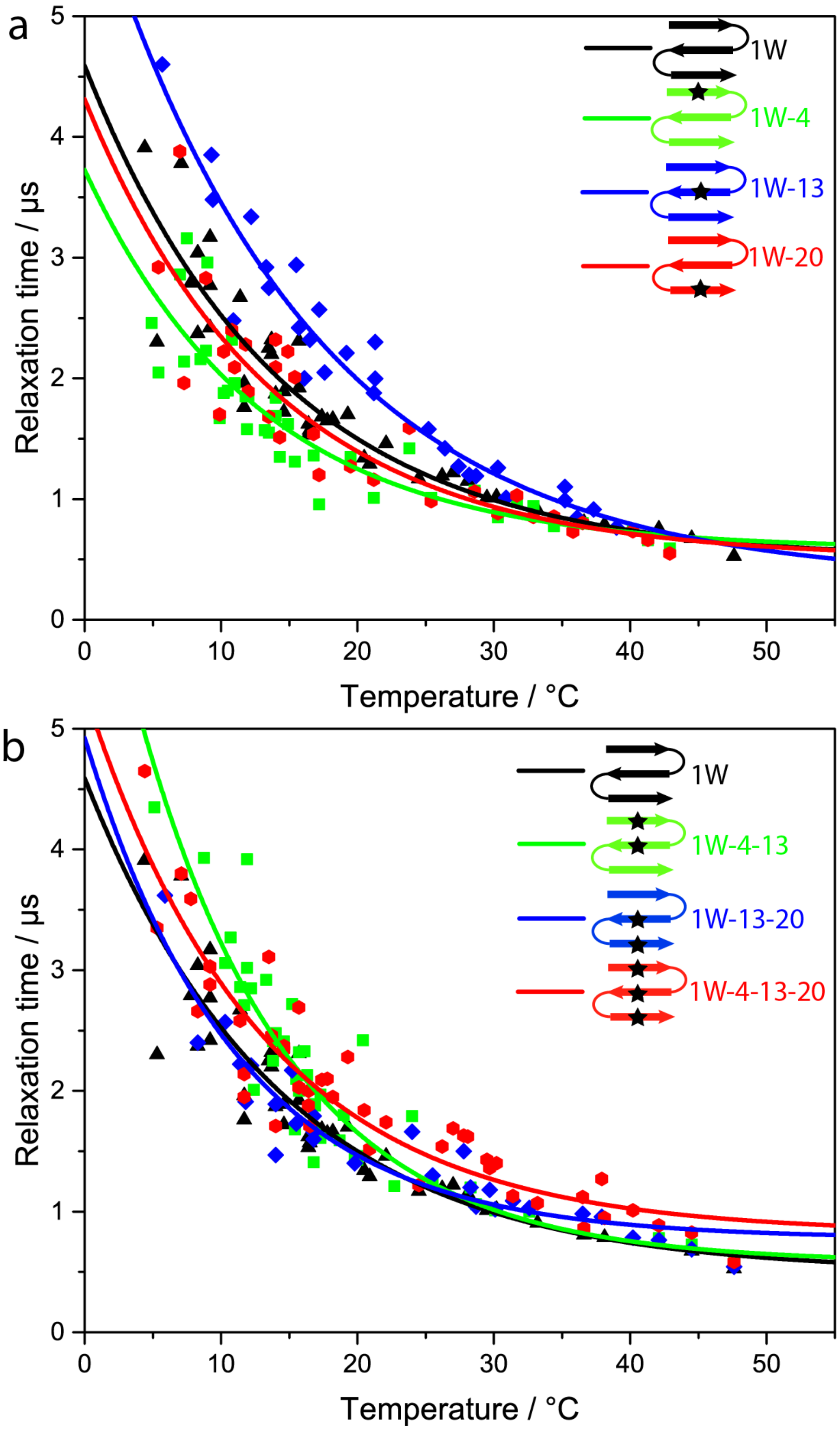

Figure S11. Comparison of the disordered component relaxation times at 1663 cm^{-1} for **a)** single labeled **1W**-variants **1W-4** (green squares), **1W-13** (blue diamonds) and **1W-20** (red circles) in comparison to the unlabeled **1W** (black triangles), and **b)** multiple labeled **1W**-variants **1W-4-13** (green squares), **1W-13-20** (blue diamonds), **1W-4-13-20** (red circles) in comparison to the unlabeled **1W** (black triangles). The lines represent fits to the Arrhenius equation as a qualitative description of the temperature dependence of the relaxation times.

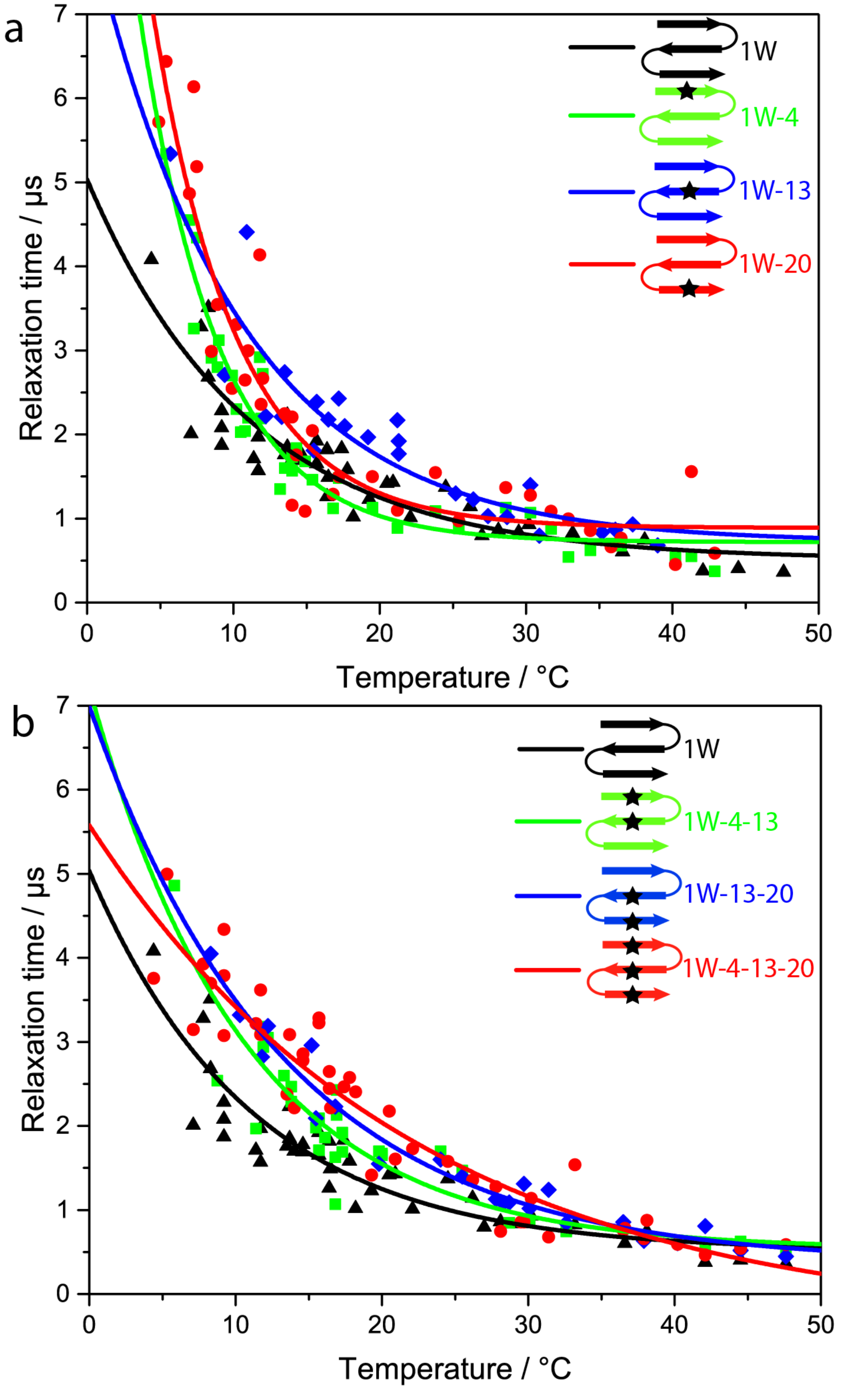

Figure S12. Comparison of the isotopic labeled bands relaxation times probed at **a)** ~1588 cm^{-1} and **b)** ~1607 cm^{-1} for **1W-4** (black triangles), **1W-20** (green squares),**1W-4-13** (blue diamonds), **1W-13-20** (red circles), **1W-4-13-20** (grey hexagons) and **1W-13** (pink triangles). The lines represent fits to the Arrhenius equation to provide a qualitative description of the temperature dependence of the relaxation times.

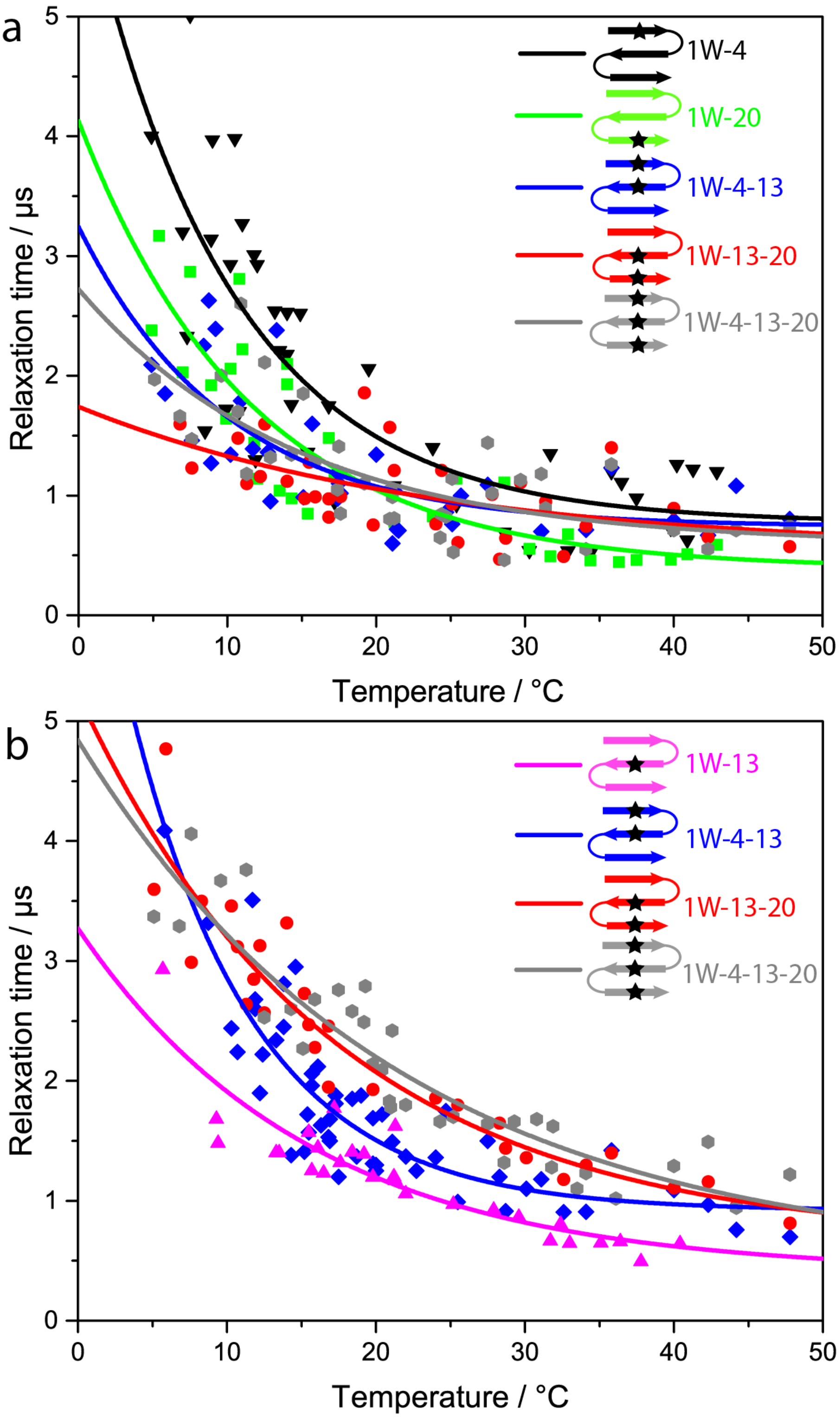

Figure S13. Relaxation times of the **1W-21** (filled symbols) as compared to those of **1W-20** (open symbols) for **a)** the $^{13}C=O$ mode, **b)** β-sheet and **c)** disordered structure. While both peptides are labeled at the C-terminal end and are adjacent residues, they show remarkable differences in the dynamics. For the $^{13}C=O$ mode, **1W-21** has a very slow relaxation. Being located so far out on the disordered C-terminal end might cause the relaxation dynamics to be dominated by the slow folding rate. Also the contribution of L21 to the overall β-sheet kinetics appears to be small, since its relaxation kinetics at 1629 cm^{-1} are hardly altered in comparison to those for **1W-20** (and therefore also the unlabeled peptide). However, the disordered kinetics are accelerated, if the slow contribution of **1W-21** is removed (see S13c).

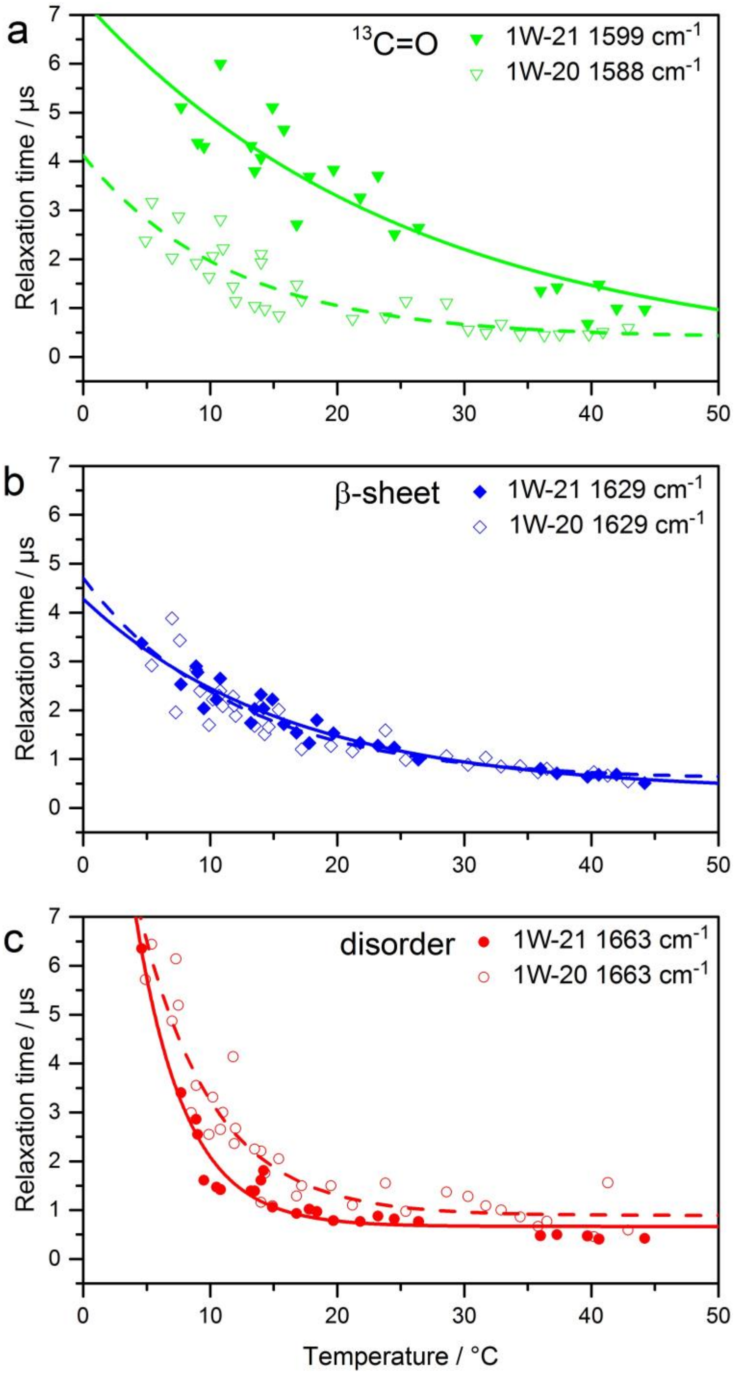

1. Frisch, M. J.; Trucks, G. W.; Schlegel, H. B; Scuseria, G. E.; Robb, M. A.; Cheeseman, J. R.; Scalmani, G.; Barone, V.; Mennucci, B.; Petersson, G. A. et al., *Gaussian 16*, Gaussian Inc.: Wallingford, CT, 2016.
2. D. Scheerer, H. Chi, D. McElheny, T. A. Keiderling and K. Hauser, *J. Phys. Chem. B*, 2018, **122**, 10445-10454.
3. P. Bouř, J. Sopková, L. Bednárová, P. Maloň and T. A. Keiderling, *J. Comput. Chem.*, 1997, **18**, 646-659.
4. Bour Laboratory, The Institute of Organic Chemistry and Biochemistry, Czech Republic. http://hanicka.uochb.cas.cz/~bour/programs/list.html (accessed June 18, 2018)).
5. R. Salomon-Ferrer, A. W. Götz, D. Poole, S. Le Grand and R. C. Walker, *J. Chem. Theory Comput.*, 2013, **9**, 3878-3888.
6. J.-P. Ryckaert, G. Ciccotti and H. J. C. Berendsen, *J. Comput. Phys.*, 1977, **23**, 327-341.
7. D. R. Roe and T. E. Cheatham, *J. Chem. Theory Comput.*, 2013, **9**, 3084-3095.
8. D. M. John and K. M. Weeks, *Protein Sci.*, 2000, **9**, 1416-1419.
